# Clinical characteristics and immune profile alterations in vaccinated individuals with breakthrough Delta SARS-CoV-2 infections

**DOI:** 10.1038/s41467-022-31693-7

**Published:** 2022-07-09

**Authors:** Qinghong Fan, Jingrong Shi, Yanhong Yang, Guofang Tang, Mengling Jiang, Jiaojiao Li, Jingyan Tang, Lu Li, Xueliang Wen, Lieguang Zhang, Xizi Deng, Yaping Wang, Yun Lan, Liya Li, Ping Peng, Yuwei Tong, Huan Lu, Lili Yan, Ying Liu, Shuijiang Cai, Yueping Li, Xiaoneng Mo, Meiyu Li, Xilong Deng, Zhongwei Hu, Haisheng Yu, Fengyu Hu, Jinxin Liu, Xiaoping Tang, Feng Li

**Affiliations:** 1 Guangzhou Eighth People’s Hospital, Guangzhou Medical University, Guangzhou, China; 2 Guangzhou Laboratory, Bio-Island, Guangzhou, China

**Keywords:** Clinical microbiology, Viral infection, SARS-CoV-2, Viral infection

## Abstract

Despite timely immunization programs, and efficacious vaccines conveying protection against SARS-CoV-2 infection, breakthrough infections in vaccinated individuals have been reported. The Delta variant of concern (VOC) outbreak in Guangzhou resulted in local transmission in vaccinated and non-vaccinated residents, providing a unique opportunity to study the protective effects of the inactivated vaccines in breakthrough infection. Here, we find that the 2-dose vaccinated group has similar peak viral titers and comparable speeds of viral RNA clearance to the non-vaccinated group but accelerated viral suppression in the middle course of the disease. We quantitatively demonstrate that peak viral pneumonia is significantly mitigated in the 2-dose vaccine group (median 0.298%) compared with the non-vaccinated (5.77%) and 1-dose vaccine (3.34%) groups. Pneumonia absorbance is approximately 6 days ahead in the 2-dose group (median 10 days) than in the non-vaccinated group (16 days) (*p* = 0.003). We also observe reduced cytokine inflammation and markedly undisturbed gene transcription profiles of peripheral blood mononuclear cells (PBMCs) in the 2-dose group. In short, our study demonstrates that prior vaccination substantially restrains pneumonia development, reduces cytokine storms, and facilitates clinical recovery.

## Abstract (web summary)

SARS-CoV-2 breakthrough infections in vaccinated individuals are a public health concern. Here, the authors analyse the clinical characteristics and profile immune alterations among vaccinated and non-vaccinated residents with Delta SARS-CoV-2 infection in Guangzhou.

## Introduction

In real-world observations, the rampant worldwide spread of SARS-CoV-2 has been curbed by timely immunization programs with multiple vaccines, which effectively elicit protective immune responses against SARS-CoV-2 infection^1–3^. The mRNA-based vaccines seem to outperform other SARS-CoV-2 vaccines in terms of protection efficacy, mainly due to their targeted delivery of viral antigen to specific antigen-presenting cells and the well-formulated and potent adjuvants that induce both T-cellular and B-cellular immunity (as has been previously reviewed^4^). However, the recent resurgence of confirmed SARS-CoV-2 infection cases has been observed globally despite the launch of massive vaccination efforts^5^. For example, the United Kingdom (UK), where 80% of the population has received two doses of an mRNA vaccine (BNT162b2 mRNA vaccine by Pfizer-BioNTech), reported a sudden increase in newly confirmed COVID-19 cases after a wave of the pandemic had subsided^6^. In Israel, where nearly 90% of the eligible population has received two doses of an mRNA vaccine since Dec 2020, a sharp decrease in reported cases was observed from the beginning of Feb 2021 to the end of Mar 2021, but a subsequent increase occurred after July 2021^7^. Even though a high portion of the population in Europe has been vaccinated, a rapid increase in newly reported cases due to Delta variant of concern (VOC) was reported in Europe^5^.

Several factors might account for this pandemic resurgence. On the one hand, breakthrough infections of the Delta VOC caused the most of these cases. All recommended vaccines, namely, the mRNA vaccines (BNT162b2 mRNA vaccine by Pfizer-BioNTech and mRNA-1273 by Moderna^8,9^), adenoviral vector vaccines (Ad26.COV2.S by Johnson & Johnson and Ad5-nCoV by Cansino Biologics^10,11^), inactivated whole viral vaccines (CoronVac by SinoVec and BBIBP-CorV by SinoPharm^12,13^), and subviral particle vaccines (ZF2001 by Anhui Zhifeng Longcom^14^), are exclusively based on the original spike protein derived from evolutionarily early SARS-CoV-2 from 2020. Accumulated mutations, such as D614G, N501Y, E484K, K417N, L452R, P681R/H, K417T, T478K, and G142D, have enhanced the viral infectivity by directly improving the binding affinity of the receptor-binding domain (RBD) to viral receptor-angiotensin converting enzyme 2 (ACE2) or contributing to neutralization escape by indirectly changing the configuration of the whole spike protein (as has been previously reviewed^15^). Delta VOC has successfully acquired several key mutations (D614G, K417N, L452R, P681R, T478K, G142D) that confer enhanced transmissibility even in the presence of neutralizing antibodies; thus, Delta VOC had become the dominant strain, accounting for 95% of newly reported cases by 30 Nov 2021 (https://nextstrain.org/ncov/gisaid/global).

On the other hand, the efficacy of vaccination over time wanes, which lowers the protective barrier against viral infection. For example, decreased protection in the long-term conferred by the BNT162b2 vaccine against the Delta variant of SARS-CoV-2 was suspected to be the leading cause of exponential growth of new SARS-CoV-2 infections in the UK^16^ and Iseral^17^. Similarly, protection induced by the mRNA-1273 vaccine against viral infection was shown to be significantly reduced 6 months post-vaccination in the USA^18^. In addition, the RBD-specific IgG elicited by inactivated vaccines decreased to marginally detectable levels in most participants 4 to 8 months following the second vaccination^19,20^. Collectively, the evidence suggests that neutralizing protection wanes over time allowing breakthrough infections of novel SARS-CoV-2 variants, necessitating a third-dose boost to reinforce the formed immunity barrier against re-infection.

Although the Delta VOC strain caused a significant increase in infected individuals globally, the patient hospitalization ratio remained stable, and no notable increase in fatal cases was reported^5^. The peak viral load has been shown to be markedly suppressed in vaccinated individuals^16^, indicating that vaccination can effectively mitigate the disease severity and reduce the mortality associated with breakthrough infections. The protective effect of the inactivated whole-virion vaccine against infection with the Delta virus has been shown to be 72.5% among participants aged 40–59 years and 100% against severe COVID-19 in a real-world setting^21^. Nevertheless, in most countries, those with COVID-19 are encouraged to stay at home unless the disease has advanced to a severe stage; thus, fewer documented clinical manifestations and laboratory characteristics have been reported for individuals with breakthrough infections. Therefore, a systematic investigation of breakthrough infections in the vaccinated population worldwide is challenging. Owing to the stringent patient management policy in China that all infected individuals should be hospitalized to receive medical care and medical supervision, investigating the changes in disease progression, lung damage, viral shedding, and cytokine profiles in real-world settings is feasible.

This study comprehensively analysed the clinical manifestations of breakthrough Delta virus infections in individuals who had received one or two doses of an inactivated vaccine. We found that two-dose vaccination protected the lungs from Delta virus attack and reduced viral-induced inflammation despite the high level of viral replication.

## Results

### Patient information

**Fig. 1:**
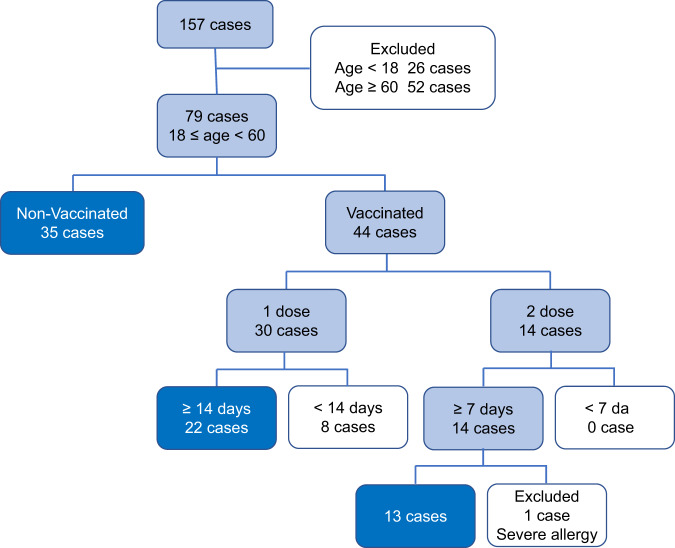
Study cohort of patients infected with SARS-CoV-2 Delta VOC. Case number and inclusion and exclusion criteria were labeled. Patients included in the analysis are highlighted. Source data are provided as a Source Data file.

In this retrospective study of an outbreak of SARS-CoV-2 Delta VOC in Guangzhou, a total of 157 patients with Delta VOC infections confirmed by the local CDC were investigated. As only individuals between 18 and 60 years old were recommended to be vaccinated before the Delta VOC outbreak in Guangzhou, 26 patients younger than 18 years and 52 patients aged 60 or older were excluded from subsequent analysis (Fig. 1). Prime immunization was assumed to take effect after 14 days. Therefore, eight patients infected within 14 days of their first vaccination were excluded. Only 14 patients received a booster immunization prior to Delta VOC infection. However, one patient was excluded due to apparent allergy prior to Delta VOC infection and throughout hospitalization. One patient infected with Delta VOC at 7 days post-booster vaccination (Supplementary Fig. 1) was also included to have adequate cases for statistical analysis in the 2-dose vaccine group. Finally, 35 non-vaccinated individuals (non-vaccinated group), 22 individuals with one shot (1-dose vaccine group), and 13 individuals with two shots (2-dose vaccine group) were included in this study.

Twelve of 35 (34%) patients were male in the non-vaccinated group, 6 of 22 (27%) were male in the 1-dose vaccine group, and 7 of 13 (53%) were male in the 2-dose vaccine group (Table 1). The median age of the non-vaccinated group was 43 (21 to 59) years old, that of the 1-dose group was 46 (21 to 58) years old, and that of the 2-dose group was 39 (27 to 58) years old. No age difference was observed between the groups. In the non-vaccinated group, 3 of 35 (9%) patients developed severe symptoms, 26 of 35 (74%) developed moderate symptoms, and 6 of 35 (17%) developed mild symptoms. In the 1-dose group, 19 of 22 (86%) patients developed moderate symptoms, and 3 of 22 (14%) developed mild symptoms. In the 2-dose group, 9 of 13 (62%) patients developed moderate symptoms, and 5 of 13 (38%) developed mild symptoms. Interestingly, the 3 patients with severe diseases in the non-vaccinated group were older (49, 53, and 59 years old) (Supplementary Fig. 2) and had hypertension, consistent with a previous study showing that age and underlying diseases were essential factors associated with disease severity^22,23^. Additionally, the distribution of clinical manifestations, including fever, cough, sputum, sore throat, dyspnea, vomiting, headache, diarrhea and fatigue, and smoking history (Table 1) were observed to be similar between the groups. Unfortunately, statistical analysis was not applicable for some cells due to small numbers. Collectively, demographic and clinical characteristic analysis failed to find significant difference between the non-vaccinated, 1-dose vaccinated, and 2-dose vaccinated patients.

**Table 1:** Demographics and baseline characteristics of COVID-19 patients infected with the Delta variant.

	Total (*N* = 70)	Non-vaccinated (*N* = 35)	1-dose vaccine (*N* = 22)	2-dose vaccine (*N* = 13)	*P* value
Demographics					
Sex, *n* (%)					
Male	25 (36)	12 (34)	6 (27)	7 (53)	0.322^a^
Female	45 (64)	23 (66)	16 (73)	6 (46)	
Age (years)					
Median (Min, Max)	44 (21, 59)	43 (21, 59)	46 (21, 58)	37 (27, 48)	0.094^b^
Clinical classification, *n*/*N* (%)					
Severe	3/70 (4)	3/35 (9)	0/22 (0)	0/13 (0)	NA
Moderate	53/70 (76)	26/35 (74)	19/22 (86)	8/13 (62)	0.208^a^
Mild	14/70 (20)	6/35 (17)	3/22 (14)	5/13 (38)	NA
Smoking history, *n*/*N* (%)	6/70 (9)	5/35 (14)	1/22 (5)	0/13 (0)	NA
Comorbidities, *n*/*N* (%)					
Hypertension	4/70 (6)	4/35 (11)	0/22 (0)	0/13 (0)	NA
Chronic heart disease	2/70 (3)	2/35 (6)	0/22 (0)	0/13 (0)	NA
Diabetes	2/70 (3)	2/35 (6)	0/22 (0)	0/13 (0)	NA
Liver disease	1/70 (1)	0/35 (0)	1/22 (5)	0/13 (0)	NA
Lung disease	1/70 (1)	0/35 (0)	1/22 (5)	0/13 (0)	NA
Thyroid disease	5/70 (7)	1/35 (3)	2/22 (9)	2/13 (15)	NA
Symptoms, *n*/*N* (%)					
Fever	55/70 (79)	30/35 (86)	17/22 (77)	8/13 (62)	NA
Cough	64/70 (91)	34/35 (97)	20/22 (91)	10/13 (77)	NA
Sputum	59/70 (84)	32/35 (91)	19/22 (86)	8/13 (62)	NA
Sore throat	30/70 (43)	16/35 (46)	10/22 (45)	4/13 (31)	0.621^c^
Dyspnea	21/70 (30)	13/35 (37)	6/22 (27)	2/13 (15)	0.377^a^
Vomit	5/70 (7)	3/35 (9)	2/22 (9)	0/13 (0)	NA
Headache	22/70 (31)	10/35 (29)	8/22 (36)	4/13 (31)	0.889^a^
Diarrhea	10/70 (14)	7/35 (20)	3/22 (14)	0/13 (0)	NA
Fatigue	17/70 (24)	12/35 (34)	4/22 (18)	1/13 (7)	0.154^a^

Statistics analysis is underpowered due to unsatisfied statistical criteria.

*NA* Not applicable.

1. Data are median (IQR), median (min, max), *n*/*N* (%) or *n* (%).

2. Two-tailed *P* values were calculated by ^a^Fisher’s exact test, ^b^ANOVA test, ^c^*χ*^2^ test, as appropriate.

### Viral replication was not affected in the upper respiratory tract

**Fig. 2:**
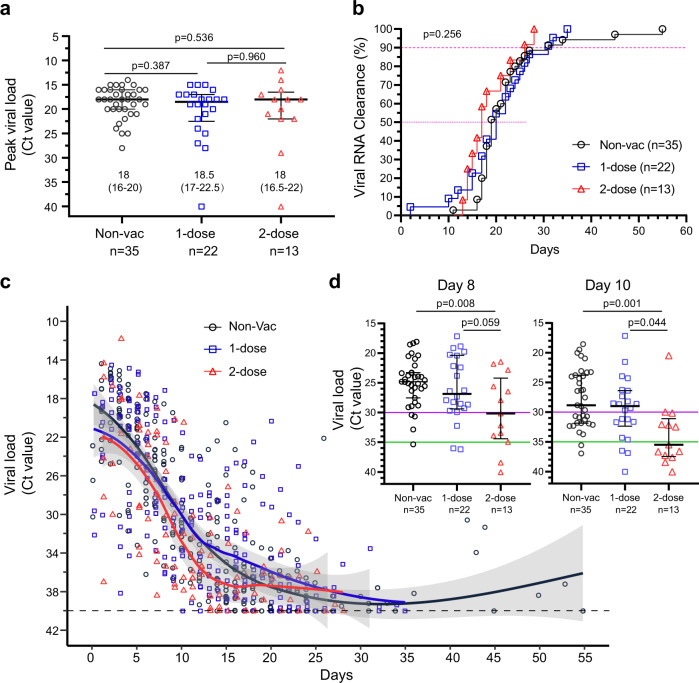
Characteristics of SARS-CoV-2 Delta VOC viral loads. **a** Peak viral load. The maximum viral load (represented by the lowest Ct value) for every patient during the entire hospitalization was selected. Two-tailed *p* values (Mann–Whitney *U* test) are indicated. Data are the median (IQR); patient numbers are labeled below each group. **b** Kinetics of SARS-CoV-2 RNA clearance. The cumulative viral clearance (percentage, %) is shown. *P* values (log-rank (Mantel–Cox) test) are indicated. **c** Overall changes in viral RNA (Ct value) during hospitalization. Each point represents one measurement. Fitted curves of the Ct value distribution are shown, smooth curves and shaded regions indicate 95% CIs. **d** Viral RNA level on day 8 and 10. Ct = 30 (purple) and Ct = 35 (green) is indicted. Data are the median (IQR); patient numbers are labeled below each group. Two-tailed *p* values (Tukey’s multiple comparisons test) are indicated. Non-Vac nonvaccinated group (black circle). 1-dose 1-dose vaccine group (blue square). 2-dose 2-dose vaccine group (red triangle). Source data are provided as a Source Data file.

We first determined whether vaccination suppressed Delta VOC replication after breakthrough infection. First, we calculated the peak viral loads (lowest PCR Ct number) from each individual and found that the median Ct values were 18 (IQR = 16–20), 18.5 (IQR = 17–22.5) and 18 (IQR = 16.5–22) for the non-vaccinated group, 1-dose vaccine group and 2-dose vaccine group, respectively (*p* > 0.05, Fig. 2a). Our results indicated that vaccination with the inactivated vaccines failed to suppress peak viral replication. Next, we analysed the viral clearance speed, defined as two consecutive viral RNA negative results over 24 h intervals. Unexpectedly, 50% of the patients in the non-vaccinated group, 1-dose group and 2-dose group exhibited viral clearance within 20, 19, and 17 days, respectively, and 90% of them exhibited viral clearance within 31, 31, and 28 days, respectively (*p* = 0.256) (Fig. 2b). Finally, all the viral titers at each time point were mapped, and we found that the trends in viral titer decline were similar between the non-vaccinated group and 1-dose vaccine group, but slightly faster in the 2-dose vaccine group (Fig. 2c). Then, we calculated the viral RNA difference from day 4 to day 14 post onset (Supplementary Table 1). Interestingly, we observed that higher portion of patients in the 2-dose vaccine group suppressed viral replication more quickly to Ct > 30 on day 8 (*p* < 0.05) and further to Ct > 35 on day 10 (*p* < 0.05) in contrast to the non-vaccinated group and 1-dose vaccine group. Finally, we compared the viral RNA loads on day 8 and 10 (Fig. 2d). The patients in the 2-dose vaccine group had much lower viral titers. Less patients in the non-vaccinated and 1-dose groups achieved fast viral suppression within 10 days. In short, our results implied that a full two-dose of vaccination only facilitated a quick viral decline in the middle course of the disease but failed to contain early viral replication and failed to clear the remaining virus in the late course of the disease.

### Two-dose vaccination substantially mitigated lung damage

**Fig. 3:**
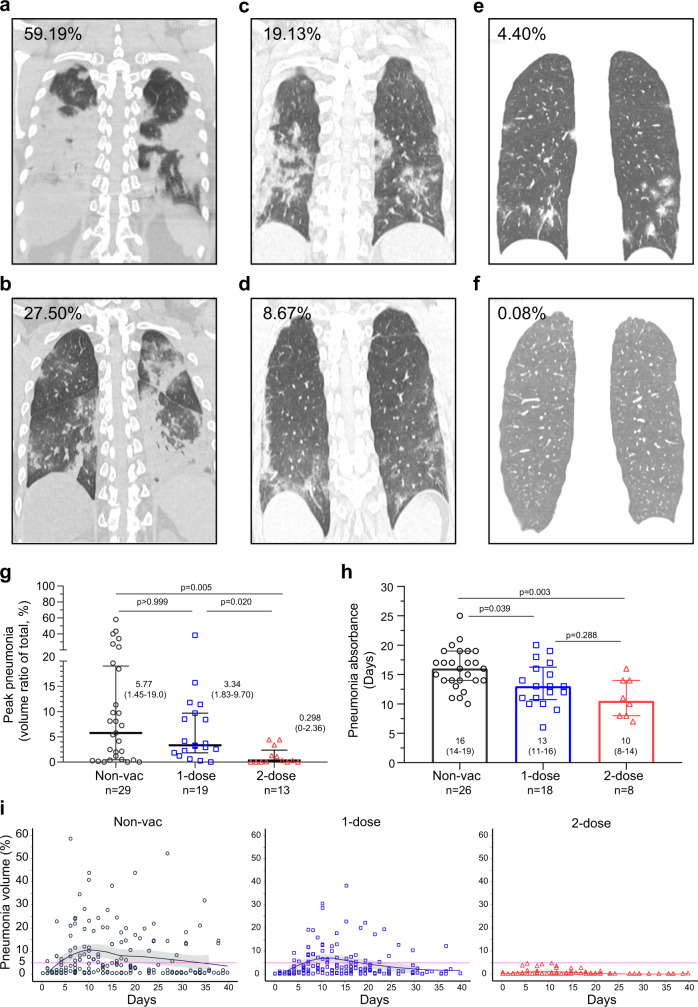
Pneumonia changes after SARS-CoV-2 Delta VOC infection. **a**–**f** Representative CT images with pneumonia volume. **a**, **b** Non-vaccinated group, (**c**, **d**) 1-dose vaccine group, (**e**, **f**) 2-dose vaccine group. **g** Peak pneumonia volume for each patient. Two-tailed *p* values (Kruskal–Wallis test) are indicated. Data are the median (IQR). **h** Pneumonia absorbance time. Two-tailed *p* values (Tukey’s multiple comparisons test) are indicated. Data are the median (IQR). **i** Overall changes in pneumonia during the whole hospitalization. Each point represents one measurement. Fitted curves of pneumonia absorbance volume distribution are shown, smooth curves and shaded regions indicate 95% CIs. Non-Vac nonvaccinated group (black circle). 1-dose 1-dose vaccine group (blue square). 2-dose 2-dose vaccine group (red triangle). Source data are provided as a Source Data file.

Another critical question was whether vaccination prevented pneumonia after Delta VOC breakthrough. Descriptive diagnoses based on chest CT imaging, such as mild or severe, unilateral or bilateral pneumonia, etc., allow direct comparison. To overcome the disadvantage of qualitative judgment, we employed quantitative measurement using the AI-based chest CT analysis model^24^. In this model, the pneumonia volume ratio of the ground-glass-like opacity versus the whole lung was accurately calculated. Six typical CT images with pneumonia volume ratios of COVID-19 caused by Delta VOC are shown (Fig. 3a–f). First, the peak pneumonia, defined as the most deteriorated lung damage, was only 0.298% (0–2.36, median ICQ) in the 2-dose vaccination group, which was substantially lower than those in the non-vaccinated group (5.77% (1.45–19.0%), *p* = 0.005) and 1-dose vaccination group (3.34% (1.83–9.70%), *p* = 0.020) (Fig. 3g). The one-dose group (3.34% (1.83–9.70%)) showed a tendency towards reduced pneumonia; however, the reduction was insignificant. Second, we analysed the time of viral pneumonia absorbance (the length of pneumonia reduced compared with earlier time points). Three patients in the non-vaccinated group, 1 in the 1-dose group and 5 in the 2-dose group, had undetectable lung pneumonia throughout the disease course. They were excluded from the subsequent pneumonia absorbance analysis. The pneumonia absorbance times were 16 (14–19, median (ICQ) days, 13 (11–16) days and 10 (8–14) days in the non-vaccinated group, one-dose group and two-dose group, respectively (Fig. 3h). The 2-dose group showed a significantly shortened pneumonia absorbance time (*p* = 0.003). One-dose vaccination also helped pneumonia absorbance (*p* = 0.039). The overall pneumonia pattern showed a notable difference between groups (Fig. 3i). In short, our results supported that 2-dose vaccination substantially mitigated pneumonia caused by Delta VOC.

### Two-dose vaccination reduced Delta virus-induced cytokine inflammation

**Fig. 4:**
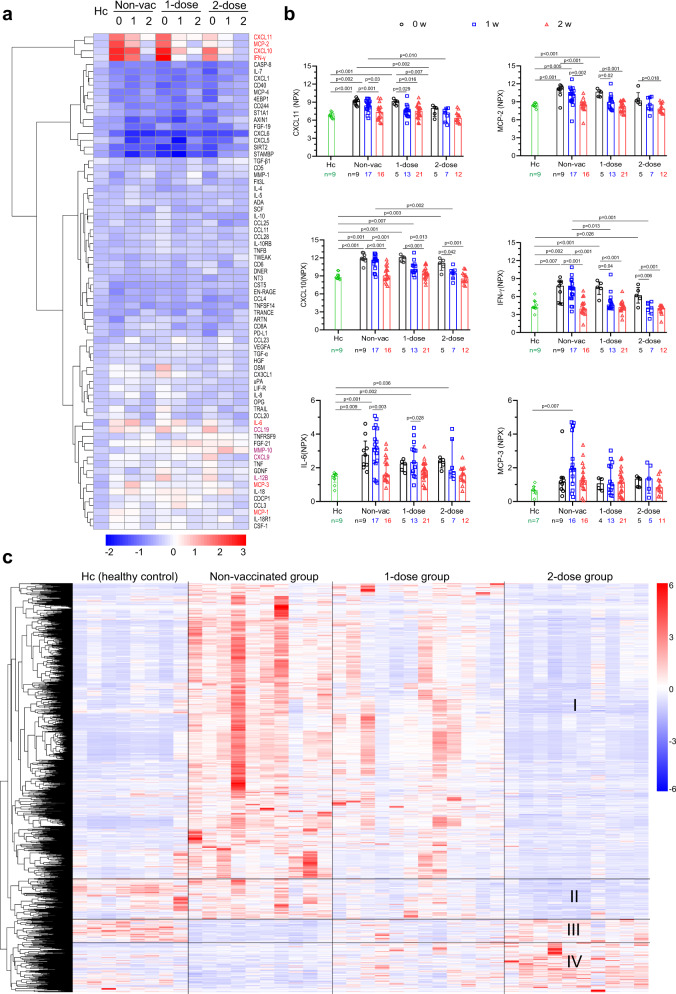
Changes in serum inflammatory cytokines and gene expression in PBMCs. **a** Serum cytokine levels at weeks 0, 1, and 2. A total of 72 (of 92) cytokines measured by O-link technology were included. **b** Kinetics of CXCL-10, CXCL11, IFN-gamma, MCP-2, IL-6, and MCP-3. NPX, normalized protein expression. Two-talied *p* values (Tukey’s multiple comparisons test) are indicated, and the data are the median (IQR). Comparisons without significant difference (*p* > 0.05) are not shown. **c** Transcriptome changes in PBMCs at week 1 (day 6–10). Heatmap showing counts per million (CPM) values of four groups based on differentially expressed genes (FDR < 0.05, |log2FC| > 1) in the nonvaccinated group versus the 2-dose group. There were 232 upregulated genes, and 1072 downregulated genes. Expression has been scaled by row (or gene). Hc healthy control. Non-Vac nonvaccinated group. 1-dose 1-dose vaccine group. 2-dose 2-dose vaccine group. 0 w week 0, days 0–5. 1 w week 1, days 6–10. 2 w week 2, days 11–16. Source data are provided as a Source Data file.

Subsequently, we analysed whether breakthrough infection resulted in hyper-inflammation, a characteristic of SARS-CoV-2 infection^25^. First, we investigated the complete changes in inflammatory cytokines using the O-link inflammation panel, which offers a simultaneous analysis of 92 protein biomarkers. Of the 92 cytokines measured, 72 passed the quality control (Fig. 4a). Compared with the healthy control, most of the cytokines were not induced. CXCL-10, CXCL11, IFN-gamma, and MCP-2 were observed to increase notably within week 0 (days 0–5) in the non-vaccinated and one-dose groups; however, their concentrations in the 2-dose group were significantly reduced (Fig. 4a, b). We also observed that CXCL6, CXCL5, SIRT2, and STAMBP decreased but not significantly. Interestingly, IL-6 and MCP-3 showed a delayed increase until week 1 (days 6–10) post-infection in the non-vaccinated group and 1-dose group. Their expression levels in the 2-dose vaccine group showed a reduced tendency (Fig. 4a, b). Inflammatory cytokine profiling indicated that vaccination markedly reduced Delta VOC-induced inflammation.

Second, we analysed the transcriptome changes in peripheral blood mononuclear cells (PBMCs) at week 1 (days 6–10, *n* = 32) after admission. One thousand differentially expressed genes were used for principal component analysis (PCA) (Supplementary Fig. 3). The 1-dose group overlapped mainly with the non-vaccinated group but was far separated from the healthy control group. However, the 2-dose group was substantially separated and was closer to the healthy control group. Then, we compared the gene expression profile in each individual (Fig. 4c). Differentially expressed genes (1034) between the non-vaccinated group and the 2-dose group were used for the heatmap. Different genes were divided into four main clusters. In the non-vaccinated group, viral infection resulted in significant activation of cluster I genes; however, those genes were essentially unchanged in the 2-dose group, close to the healthy control group. The cluster II genes seemed unchanged in the non-vaccinated group, but they were downregulated after viral infection in both the 1-dose and 2-dose groups. The cluster III genes were visually downregulated in the non-vaccinated and 1-dose groups but were unaltered in the 2-dose group. Cluster IV genes were upregulated in the 2-dose group, compared with the healthy control and nonvaccinated groups. Gene set enrichment analysis (GSEA) showed that the innate immune response and interferon pathways were downregulated (Supplementary Fig. 4) In summary, our results implied that 2-dose vaccination suppressed viral-induced inflammation.

### Breakthrough infection promptly recalled the humoral immune response elicited by vaccination

**Fig. 5:**
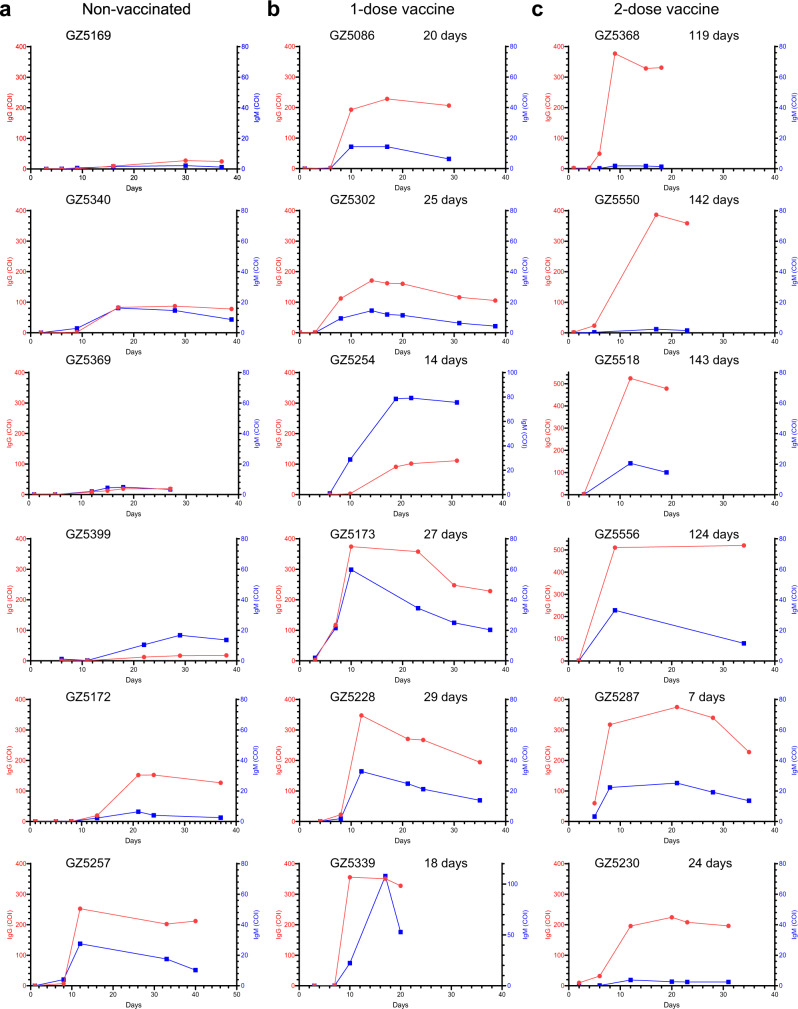
Features of anti-RBD-specific IgM and IgG. **a**–**c** Anti-RBD-specific IgM and IgG from representative individuals in the nonvaccinated group (**a**), in 1-dose vaccine group (**b**), and in the 2-dose vaccine group (**c**). Patient number and interval from last vaccination to infection are indicated on top of each individual. The red circle represents RBD-IgG. The blue square represents RBD-IgM. COI cut-off index. Source data are provided as a Source Data file.

Next, we examined viral-specific antibody generation in Delta VOC-infected individuals. The IgM and IgG titers were collected from the laboratory information system. Expectedly, breakthrough infection resulted in instantly higher RBD-specific IgM and IgG (Supplementary Fig. 5). Breakthrough infection first rapidly recalled viral-specific memory. On average, it took approximately nine days before RBD-specific IgM and IgG became positive (>1 COI) in the non-vaccinated group. Both vaccinated groups had higher background levels of RBD-specific IgM and IgG; therefore, their antibody titers rose rapidly within 1 week. Then, we presented the antibody changes in representative individuals (Fig. 5 and Supplementary Fig. 6). Of all 35 patients in the non-vaccinated group, only one individual (patient GZ5257) exhibited a high level of RBD-IgG (>200 COI) (Fig. 5a). In contrast, the RBD-IgG increased quickly from barely detected to over 300 COI in some individuals (GZ5173, GZ5228, GZ5339, GZ5288) in the 1-dose vaccine group (Fig. 5b and Supplementary Fig. 6b). Patients (GZ5518 and GZ5556) with the 2-dose vaccine produced extremely high levels of IgG in (>500 COI), and the majority of the patients had high levels of RBD-IgG (>200 COI) (Fig. 5c and Supplementary Fig. 6c). Notably, four individuals (GZ5368, GZ5550, GZ5518 and GZ5556) with only had marginal levels of RBD-IgG when infected 4 months after the last vaccination (119, 142, 143, and 124 days, respectively) exhibited the highest levels of RBD-IgG (360 to 520 COI). In short, our study indicated that viral-specific antibodies increased promptly in the vaccinated groups.

### Two-dose vaccination prevented over-activation of the immune activation

**Fig. 6:**
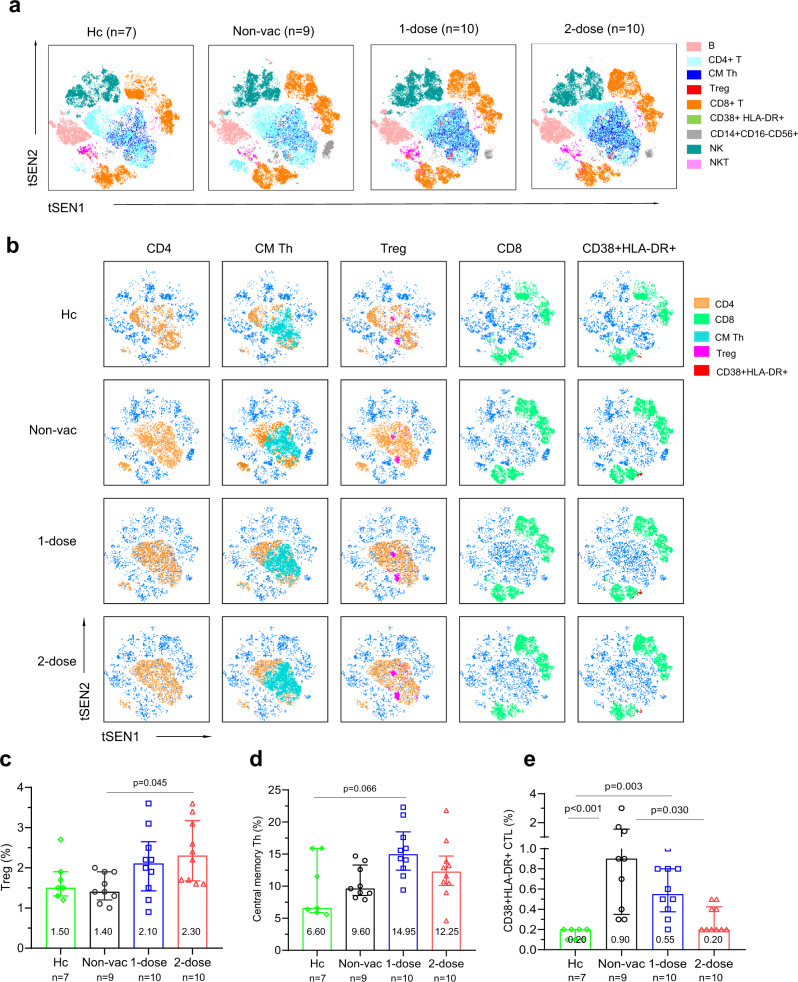
Flow cytometry analysis of the lymphocyte subsets. **a** T-SNE plot of circulating lymphocyte subsets in the healthy control (*n* = 7), nonvaccinated (*n* = 9), 1-dose vaccine (*n* = 10) or 2-dose vaccine (*n* = 10) groups; pooling from 10,000 cellular events in each sample based on 24 surface markers of spectral flow cytometric analysis. Cells are colored according to different subsets. **b** T-SNE plot of lymphocyte subsets with significant differences among the four groups, including helper T (Th) cells (CD3+CD4+), central memory Th (CM Th) cells (CD3+CD4+CD45RA-CCR7+), regulatory T (Treg) cells (CD3+CD4+CD25+CD127low), cytotoxic T lymphocytes (CTL, CD3+CD8+) and CD38+HLA-DR+CTLs (CD3+CD8+CD38+HLA-DR+). **c**–**e** Percentages of Treg cells (**c**), CM Th cells (**d**) and CD38+HLA-DR+CTLs (**e**) within circulating leukocytes are shown. Adjusted *p* values (Kruskal–Wallis test) are indicated in the figure, and data are shown as the median (IQR). Non-Vac nonvaccinated group. 1-dose 1-dose vaccine group. 2-dose 2-dose vaccine group. Source data are provided as a Source Data file.

Finally, we analysed the composition and activation status of PBMCs using 24-color flow cytometry. The gating strategies are illustrated (Supplementary Figs. 7, 8). Frozen samples (days 6–10 post-admission) from the healthy control group (*n* = 7), non-vaccinated group (*n* = 9), 1-dose vaccine group (*n* = 10) and 2-dose vaccine group (*n* = 10) were used. Flow cytometry populations were resolved and clustered using tSEN (Fig. 6a). An overall visualization of all the cell populations implied that central memory T helper cells (CM Th), T regulatory cells (Treg), activated CD8 T cells (CD3+CD8+CD38+HLA-DR+) and natural killer cells (NK, CD3-CD56+) differed among the four groups (Fig. 6a, b). Treg cells were significantly higher in the 2-dose group (median, 2.30) than in the healthy control (1.50) and the non-vaccinated groups (1.40) (*p* = 0.045) (Fig. 6c). A similar tendency was observed in the 1-dose group (2.10), but the difference was not significant. Central memory Th cells increased in both the 1-dose (14.95) and 2-dose (12.25) groups (Fig. 6d). The percentage of CD8+CD38+HLA-DR+T cells increased substantially in the nonvaccinated group (0.90%) and 1-dose group (0.55%) compared with the healthy control group (0.20) (*p* < 0.001 and *p* = 0.003, respectively) (Fig. 6e). This population decreased to a normal level in the 2-dose group (0.20%). However, the NKT and CD14+CD56+ cell populations were not significantly different (Supplementary Fig. 9). The difference observed in tSEN (Fig. 6a) might be due to the exceptional outliner. In summary, the immune population changes among the different groups indicated that preexisting viral-specific memory in the 2-dose vaccinated group might still play a vital role in regulating the immune response against Delta VOC breakthrough infections.

## Discussion

To our knowledge, this is the first systematic analysis of the changes in pneumonia, laboratory tests, and immune profiles associated with a heterologous SARS-CoV-2 (Delta variant) breakthrough infection in individuals who received an inactivated prototype SARS-CoV-2 strain vaccines. The SARS-CoV-2 virus has changed substantially since its emergence in humans at the end of 2019. Multiple variants are cocirculating in most regions globally^26,27^. Even in the same strain, the dominant Delta VOC has acquired more mutations and has been divided into different sublineages. One major advantage of this study is that all the included patients were confirmed both epidemiologically and via sequencing to be infected with one single strain^28^. Thus, we did not have to consider the mixed effect of different SARS-COV-2 stains and lineages in our cohort. In addition, we still have nonvaccinated control in our study. Only some individuals were vaccinated with either one or two doses when Delta VOC first broke out in Guangzhou. After this outbreak, however, almost all the eligible individuals were rapidly vaccinated nationally. Thus, it became challenging to include a sufficient number of non-vaccinated individuals. Therefore, our study provides a valuable opportunity to investigate clinical manifestations and vaccine protection when breakthrough infections occur.

Most importantly, we found that two-dose vaccination significantly reduced viral-induced symptoms as evidenced by (1) notable pneumonia mitigation and shortened pneumonia absorbance time evaluated by CT imaging, (2) significant subsidence of serum cytokine inflammation, and (3) nearly normal gene expression profiles in blood PBMCs. First, as the lung is the primary organ for SARS-COV-2 infection, reducing lung damage prevents disease development to severe and critical stages and saves patients’ lives. CT imaging examination can accurately detect lung damage. One typical characteristic of SARS-CoV-2 pneumonia is the ground-glass opacity^24^, which substantially differs from other non-COVID-19 pneumonia in the lung^29,30^. Generally, identification and determination of lesions of ground-glass opacity by experts provide only a descriptive quantification, such as unilateral or bilateral, spotty or continuous, low- or high-grade damage^29,30^. Artificial-intelligence-based computed tomography (CT) chest imaging analysis, however, can not only identify viral-induced lesions but also provide quantitative calculations, allowing for the direct comparison of pneumonia severity^24,31,32^. In this study, receiving 2 doses of an inactivated vaccine significantly curtailed pneumonia spread after Delta VOC breakthrough, limited the volume to under 5% throughout the entire disease course, and accelerated lung recovery (6 days ahead of nonvaccinated patients) (Fig. 3g–i). One-dose vaccination also promoted pneumonia absorbance (Fig. 3h). Second, the magnitude of inflammation, which is usually correlated with pneumonia severity, was extensively evaluated^33,34^. By simultaneously measuring the concentration of 92 inflammatory cytokines, we demonstrated that 2-dose vaccination substantially decreased inflammation (Fig. 4a, b). The difference would have become more significant if we had more severe patients in the non-vaccinated group. Finally, the transcriptome profile of the 2-dose vaccine group was much closer to that of the healthy control group (Fig. 4c, Supplementary Fig. 3). Therefore, 2-dose vaccines effectively prevented pneumonia development and significantly reduced viral-induced inflammation.

Our study had several limitations. First, the patient cohort did not include all ages with underlying diseases. All the patients were between the ages of 18 and 59 years. Only after this Delta VOC outbreak in Guangzhou did the aged population (60 years and older) and adolescents (10 < age < 18) become massively vaccinated. In addition, the aged population tended to have multiple underlying diseases, such as diabetes and hypertension. We do not know whether 2-dose vaccines would protect the older population from developing severe symptoms in breakthrough infections. Adolescents and children are more likely to have mild disease and were not vaccinated for safety concerns at the trial stage of massive inactivated vaccine application. Three patients developed severe symptoms in the non-vaccinated group; however, two were ~50 years old and had hypertension (Supplementary Fig. 1). There were no patients with underlying disease in the vaccinated cohorts. Second, the prediction that high titers of viral-specific antibodies would be rapidly generated in patients long after vaccination when antibodies waned is still preliminary. We had only four patients in the 2-dose group infected 4–5 months post-vaccination. If we had more breakthrough cases spanning a wide follow-up time post-vaccination (for example, 1–8 months), we would have the opportunity to examine whether long-term vaccine memory maintains a prompt reaction to another infection. Third, the cause of the discrepancy between significantly alleviated pneumonia in the lung and high and persistent viral RNA in the upper respiratory tract in the 2-dose vaccine group is unknown. We speculated that Delta VOC escaped protection at a low level of protective antibodies but stimulated vaccine-specific memory humoral and cellular immune responses. Viral-specific adaptive immunity in the lung can prevent pneumonia development but cannot effectively control viral replication in the upper respiratory tract. Unfortunately, we had no lung fluid samples for viral RNA analysis. Finally, the viral-specific T-cellular immune response was absent because fresh PBMCs from the patients were unavailable. Serum neutralization against Delta VOC in the late stage of breakthrough infections was not analysed due to a shortage of high-level biological safety laboratory facilities.

Taken together, our intensive investigation of Delta VOC breakthrough infections suggested that prior vaccination with the inactivated vaccine can significantly mitigate pneumonia caused by a heterologous viral infection and reduced viral-induced inflammation, and implied that the current vaccination program might work in protecting the whole population from developing severe symptoms when infected with other heterologous SARS-CoV-2 strains.

## Methods

### Participants

All patients were from Guangzhou Eighth People’s Hospital, Guangzhou Medical University from May 21, 2021 (the first case was officially confirmed to be infected with SARS-CoV-2 Delta VOC) to July 9, 2021 (the discharge day of all local patients). All patients were confirmed to be linked to a single Delta VOC origin by epidemiological evidence and viral genome sequencing^28^. A total of 157 individuals were included in this study. The study was approved by Guangzhou Eighth People’s Hospital Ethics Committee (No. 202001134 and 202115202). Written informed consents were obtained from all patients.

### Clinical data collection

Clinical characteristic, laboratory findings were collected from the hospital information system and laboratory information system of the hospital. All diagnoses were made based on the Guidelines for the Diagnosis and Treatment of Novel Coronavirus Infection produced by the Chinese National Health Commission (Trial Version 8).

Severe patients here refer to both severe and critically ill patients meeting any of the following:

1. Shortness of breath, respiratory rate ≥30 times/min;
2. In the resting state, during inhalation, the oxygen saturation is ≤93%;
3. Arterial partial pressure of oxygen (PaO2)/inhaled oxygen concentration (FiO2) ≤300 mmHg (1 mmHg = 0.133 kPa);
4. The clinical symptoms are progressively worsening, and lung imaging shows that within 24 to 48 h the lesion has progressed significantly >50%.

The moderate symptom was diagnosed when patients had visible pneumonia, and fever, and/or other respiratory symptoms. The Mild symptom was diagnosed when slight uncomforted but without pneumonia symptoms. Asymptomatic cases were diagnosed when the patients had no sign of clinical symptoms but were confirmed to be SARS-CoV-2 viral RNA positive during the follow-up stage after viral exposure or close contact with confirmed cases.

Clinical characteristic included clinical classification (mild, moderate, severe), comorbidities (hypertension, diabetes, chronic heart disease, liver disease, lung disease, and thyroid) and symptoms (fever, cough, sputum, sore throat, dyspnea, vomit, headache, diarrhea, fatigue.). Demographics included sex, age, smoking history.

### Viral RNA detection with RT-PCR

The nasopharyngeal swabs were collected by well-trained medical staff following the standardized procedures. The samples in virus medium were stored and transferred into the lab with 2 h before viral measurement. Viral RNA was extracted using the Nucleic Acid.

Isolation Kit (Da’an Gene Co. Ltd, Cat: DA0630, China) and the subsequent RT-PCR detection was performed by using the RNA Detection Kit for SARS-CoV-2 (Da’an Gene Co. Ltd, Cat: DA0930, China) according to the manufacturer’s instructions^35^. RT-PCR was conducted with primers and probes targeting at the N, ORF1a/b genes and a positive reference gene. The detection limit of cycle threshold (Ct) was set to be 40 (500 copies/ml). Samples with Ct of less than 40 were considered positive. All tests were performed under strict biosafety conditions and the standard operating procedures.

### Lung damage calculation computed tomography (CT) imaging

The lung damage was calculated by an artificial-intelligence model developed in Guangzhou Eighth People’s Hospital^24^. Briefly, the chest CT imaging data set from SARS-VoV-2 viral RNA confirmed patients were collected from the hospital information system and were subject to two-step calculations. The AI model would first detect any abnormality in every single slice and discriminate which lesion were caused by the viral infection; then it summed up the value of the continuous lesion region to generate the volume ratio (VI ratio) of the lesion in the left and the right lung. The volume of left lung (46.5%) and right lung (53.5%) were adjusted according to a previous study^36^. Total volume ratio (VR) = 46.5% × left *VR* + 53.5% × right *VR*. The calculations were also confirmed by experienced radiologists.

### Antibody detection (measurement of RBD-specific IgG and IgM antibodies.)

Plasma samples were inactivated at 56 °C for 30 min before testing. IgG and IgM antibodies against the SARS-CoV-2 RBD spike protein were tested with two-step indirect immunoassay electrochemiluminescence immunoassay kits (Antu Biotech Co., Ltd.), according to the manufacturer’s instructions. Briefly, the samples were first incubated with microparticles coated with the RBD of the SARS-CoV-2 spike protein and acridine ester-labeled antibodies against the Fc domain of human antibodies. After the unbound substances were washed off, signal detection was performed on an automatic chemiluminescence immunoanalyzer (AutoLumo A1000, Antu Biotech Co., Ltd.). All tests were performed under strict biosafety conditions.

### Cytokine measurement

Inflammation multiplex platform (Cat. v.3022, Olink Proteomics AB, Uppsala, Sweden) including 92 inflammation related protein biomarkers were used in this study (https://www.olink.com/products-services/target/inflammation/). The OLINK immunoassays are based on the Proximity Extension Assay technology. When a pair of oligonucleotide-labeled antibodies bind to their respective target protein and docked closely, the oligonucleotide attached to each antibodies anneals, which provide template and primer for initiating a new polymerase chain reaction (PCR). Then, the PCR product were used to generate library in which the concentration of each product is quantitated by next generation sequencing. Finally, the protein concentration is calculated basing on the concentration of PCR product.

### RNA isolation, library construction, and sequencing

Whole PBMCs (ficoll prepared, storaged in liquid nitrogen before) of each patient and healthy donors were revived with RPMI 1640 medium (GIBCO, 11875093) containing 10% fetal bovine serum (GIBCO, 10270-106), then washed once with DPBS (BI, 02-023-1A) immediately. Total RNA was isolated using Trizol Reagent (Invitrogen, 15596018), purified by mRNA Capture Beads (VAHTS, N401-02), and RNA library construction were performed following the manufacturer’s instructions (MGIEasy RNA Library Prep Kit, 1000006383). Qualified RNA library was sequenced with MGISEQ-2000 platform (MGI, Shenzhen, PR China) in a 100 double-end sequencing method.

### RNA-sequencing data processing and analysis

Raw reads were trimmed with fastq and mapped to human hg38 genome using STAR. Raw counts were obtained with featureCounts, then filtered to remove those genes with an extremely low expression. Differential expression analysis and CPM were performed using edgeR (v.3.32.1) with “TMM” method. The metric score for GSEA is the sign of fold change multiplied by −log_10_ *P* value. GSEA were generated with fgsea (v.1.16.0) using BTMs as pathways.

### Flow cytometry

Multi-parametric flow cytometry was used for phenotypic analysis of PBMCs from patients and healthy donors. Briefly, 1 × 106–2 × 106 PBMCs were washed twice with DPBS (BI, 02-023-1A) and stained with FVS575V for 20 min at RT in the dark for live/dead. Then cells were washed twice (400 × *g*, 5 min, 4 °C) with FACS buffer (2% FBS/PBS) and stained with CXCR5-BV650 and CCR7-BV421 for 10 min at RT in the dark. Next, other 21 kinds of surface receptor staining mix were incubated with cells for another 30 min at RT in the dark. After incubation, cells were washed with FACS buffer (400 × *g*, 5 min, 4 °C) and resuspended in 200 μl FACS buffer. The flow cytometric data were collected on a spectral flow cytometry (Cytek NL-CLC, Cytek Biosciences, USA) and analyzed using FlowJo software, V10.7.1 (Tree Star, USA). Detailed information of antibodies was shown in Supplementary Table 2.

### Statistics analysis

Continuous variables were expressed as median (interquartile range, IQR). Categorical variables were summarized as the counts and percentages in each category. Mann–Whitney *U* tests, ANOVA tests or Kruskal–Wallis tests were applied to continuous variables as appropriate, chi-square test or Fisher’s exact test were applied to categorical variables as appropriate, log-rank (Mantel–Cox) test was applied to virus RNA clearance, *p* < 0.05 was considered statistically significant. Statistical analysis was performed with IBM SPSS Statistics 25. Graphic representations were performed with GraphPad Prism 8.0.1 software. Full-spectrum heatmap of 92 inflammatory cytokines were performed with pheatmap packages of R studio. RNA-sequencing data processing and analysis were performed with fastp, STAR, featureCounts, edgeR (v.3.32.1) packages, fgsea (v.1.16.0) packages. The flow cytometric data were analyzed using FlowJo software V10.7.1.

### Reporting summary

Further information on research design is available in the Nature Research Reporting Summary linked to this article.

## Supplementary information

Supplementary Information

Reporting Summary

## Data Availability

The raw RNA-sequencing data reported in this paper have been deposited in the Genome Sequence Archive for Human under accession code HRA002352 and are accessible at https://ngdc.cncb.ac.cn/gsa-human/s/S0u3Oey2. Source data are provided with this paper.

## Source data

Source Data

## References

[1] Self WH (2021). Comparative Effectiveness of Moderna, Pfizer-BioNTech, and Janssen (Johnson & Johnson) Vaccines in Preventing COVID-19 Hospitalizations Among Adults Without Immunocompromising Conditions—United States, March–August 2021. MMWR Morb. Mortal. Wkly Rep..

[2] Haas EJ (2021). Impact and effectiveness of mRNA BNT162b2 vaccine against SARS-CoV-2 infections and COVID-19 cases, hospitalisations, and deaths following a nationwide vaccination campaign in Israel: an observational study using national surveillance data. Lancet.

[3] Angel Y (2021). Association between vaccination with BNT162b2 and incidence of symptomatic and asymptomatic SARS-CoV-2 infections among health care workers. JAMA.

[4] Bian L (2021). Impact of the Delta variant on vaccine efficacy and response strategies. Expert Rev. Vaccines.

[5] WHO. WHO Coronavirus (COVID-19) Dashboard. https://covid19.who.int/?mapFilter=vaccinations (2021).

[6] Government, UK. The official UK government website for data and insights on coronavirus (COVID-19). https://coronavirus.data.gov.uk/details/vaccinations (2021).

[7] Goldberg Y (2021). Waning immunity after the BNT162b2 vaccine in Israel. N. Engl. J. Med.

[8] Baden LR (2021). Efficacy and safety of the mRNA-1273 SARS-CoV-2 vaccine. N. Engl. J. Med.

[9] Polack FP (2020). Safety and efficacy of the BNT162b2 mRNA Covid-19 vaccine. N. Engl. J. Med.

[10] Sadoff J (2021). Safety and efficacy of single-dose Ad26.COV2.S vaccine against Covid-19. N. Engl. J. Med.

[11] Zhu FC (2020). Safety, tolerability, and immunogenicity of a recombinant adenovirus type-5 vectored COVID-19 vaccine: a dose-escalation, open-label, non-randomised, first-in-human trial. Lancet.

[12] Xia S (2021). Safety and immunogenicity of an inactivated SARS-CoV-2 vaccine, BBIBP-CorV: a randomised, double-blind, placebo-controlled, phase 1/2 trial. Lancet Infect. Dis..

[13] Zhang Y (2021). Safety, tolerability, and immunogenicity of an inactivated SARS-CoV-2 vaccine in healthy adults aged 18-59 years: a randomised, double-blind, placebo-controlled, phase 1/2 clinical trial. Lancet Infect. Dis..

[14] Yang S (2021). Safety and immunogenicity of a recombinant tandem-repeat dimeric RBD-based protein subunit vaccine (ZF2001) against COVID-19 in adults: two randomised, double-blind, placebo-controlled, phase 1 and 2 trials. Lancet Infect. Dis..

[15] Tian D, Sun Y, Zhou J, Ye Q (2021). The global epidemic of SARS-CoV-2 variants and their mutational immune escape. J. Med Virol..

[16] Elliott P (2021). Exponential growth, high prevalence of SARS-CoV-2, and vaccine effectiveness associated with the Delta variant. Science.

[17] Mizrahi B (2021). Correlation of SARS-CoV-2-breakthrough infections to time-from-vaccine. Nat. Commun..

[18] Pegu A (2021). Durability of mRNA-1273 vaccine-induced antibodies against SARS-CoV-2 variants. Science.

[19] Ju B (2022). Potent antibody immunity to SARS-CoV-2 variants elicited by a third dose of inactivated vaccine. Clin. Transl. Med..

[20] Ai J (2021). Recombinant protein subunit vaccine booster following two-dose inactivated vaccines dramatically enhanced anti-RBD responses and neutralizing titers against SARS-CoV-2 and Variants of Concern. Cell Res..

[21] Li XN (2021). Effectiveness of inactivated SARS-CoV-2 vaccines against the Delta variant infection in Guangzhou: a test-negative case-control real-world study. Emerg. Microbes Infect..

[22] Williamson EJ (2020). Factors associated with COVID-19-related death using OpenSAFELY. Nature.

[23] Zhou F (2020). Clinical course and risk factors for mortality of adult inpatients with COVID-19 in Wuhan, China: a retrospective cohort study. Lancet.

[24] Yang Y (2021). Using artificial intelligence to assist radiologists in distinguishing COVID-19 from other pulmonary infections. J. Xray Sci. Technol..

[25] Long QX (2020). Clinical and immunological assessment of asymptomatic SARS-CoV-2 infections. Nat. Med.

[26] Li J, Lai S, Gao GF, Shi W (2021). The emergence, genomic diversity and global spread of SARS-CoV-2. Nature.

[27] van Dorp CH, Goldberg EE, Hengartner N, Ke R, Romero-Severson EO (2021). Estimating the strength of selection for new SARS-CoV-2 variants. Nat. Commun..

[28] Wang Y (2021). Transmission, viral kinetics and clinical characteristics of the emergent SARS-CoV-2 Delta VOC in Guangzhou, China. EClinicalMedicine.

[29] Guan W, Liu J, Yu C (2020). CT findings of coronavirus disease (COVID-19) severe pneumonia. AJR Am. J. Roentgenol..

[30] Xu X (2020). Imaging and clinical features of patients with 2019 novel coronavirus SARS-CoV-2. Eur. J. Nucl. Med. Mol. Imaging.

[31] Wang B (2021). AI-assisted CT imaging analysis for COVID-19 screening: building and deploying a medical AI system. Appl. Soft. Comput.

[32] Harmon SA (2020). Artificial intelligence for the detection of COVID-19 pneumonia on chest CT using multinational datasets. Nat. Commun..

[33] Bonnet B (2021). Severe COVID-19 is characterized by the co-occurrence of moderate cytokine inflammation and severe monocyte dysregulation. EBioMedicine.

[34] Huang C (2020). Clinical features of patients infected with 2019 novel coronavirus in Wuhan, China. Lancet.

[35] Hu F (2020). A compromised specific humoral immune response against the SARS-CoV-2 receptor-binding domain is related to viral persistence and periodic shedding in the gastrointestinal tract. Cell Mol. Immunol..

[36] Yamada Y (2020). Differences in lung and lobe volumes between supine and standing positions scanned with conventional and newly developed 320-detector-row upright CT: intra-individual comparison. Respiration.

