# Publisher Correction: Clinical characteristics and immune profile alterations in vaccinated individuals with breakthrough Delta SARS-CoV-2 infections

**DOI:** 10.1038/s41467-022-32734-x

**Published:** 2022-09-06

**Authors:** Qinghong Fan, Jingrong Shi, Yanhong Yang, Guofang Tang, Mengling Jiang, Jiaojiao Li, Jingyan Tang, Lu Li, Xueliang Wen, Lieguang Zhang, Xizi Deng, Yaping Wang, Yun Lan, Liya Li, Ping Peng, Yuwei Tong, Huan Lu, Lili Yan, Ying Liu, Shuijiang Cai, Yueping Li, Xiaoneng Mo, Meiyu Li, Xilong Deng, Zhongwei Hu, Haisheng Yu, Fengyu Hu, Jinxin Liu, Xiaoping Tang, Feng Li

**Affiliations:** 1 Guangzhou Eighth People’s Hospital, Guangzhou Medical University, Guangzhou, China; 2 Guangzhou Laboratory, Bio-Island, Guangzhou, China

**Keywords:** Clinical microbiology, Viral infection, SARS-CoV-2, Viral infection

Correction to: *Nature Communications* 10.1038/s41467-022-31693-7, published online 09 July 2022

In this article Guofang Tang, Mengling Jiang and Jiaojiao Li should have been denoted as equally contributing authors. The original article has been corrected.

