# Supplementary material for: Clinical characteristics and immune profile alterations in vaccinated individuals with breakthrough Delta SARS-CoV-2 infections
Source: Nat Commun. 2022 Jul 9;13:3979. doi: 10.1038/s41467-022-31693-7 (PMC9271076; doi:10.1038/s41467-022-31693-7)
Supplement: Supplementary file 1 — Supplementary Information [file 41467_2022_31693_MOESM1_ESM.pdf]

**Supplementary information of**

Title: **Clinical characteristics and immune profile alterations in vaccinated individuals with breakthrough Delta SARS-CoV-2 infections**

Supplementary Figures: 9

Supplementary Tables: 2

**Supplementary Figure 1.** Time of breakthrough infection post-vaccination.

**Supplementary Figure 2.** Age, clinical classification, and comorbidity distribution.

**Supplementary Figure 3.** Principal component analysis of CPM values from transcriptome data.

**Supplementary Figure 4.** Gene set enrichment analysis.

**Supplementary Figure 5.** Trend of anti-RBD-specific IgM and IgG during the entire admission.

**Supplementary Figure 6.** Features of Anti-RBD-specific IgM and IgG.

**Supplementary Figure 7.** Gating logic of multi-parametric flow cytometry.

**Supplementary Figure 8.** Staining control of multi-parametric flow cytometry.

**Supplementary Figure 9.** Flow cytometry analysis of CD14+CD56+NK, NKT, and CD14+ mean fluorescence intensity (MFI).

**Supplemental Table 1.** Viral RNA level comparison from day 4 to day14 post symptom onset.

**Supplemental Table 2.** Antibodies used for flow cytometry analysis.

Supp. Figure 1

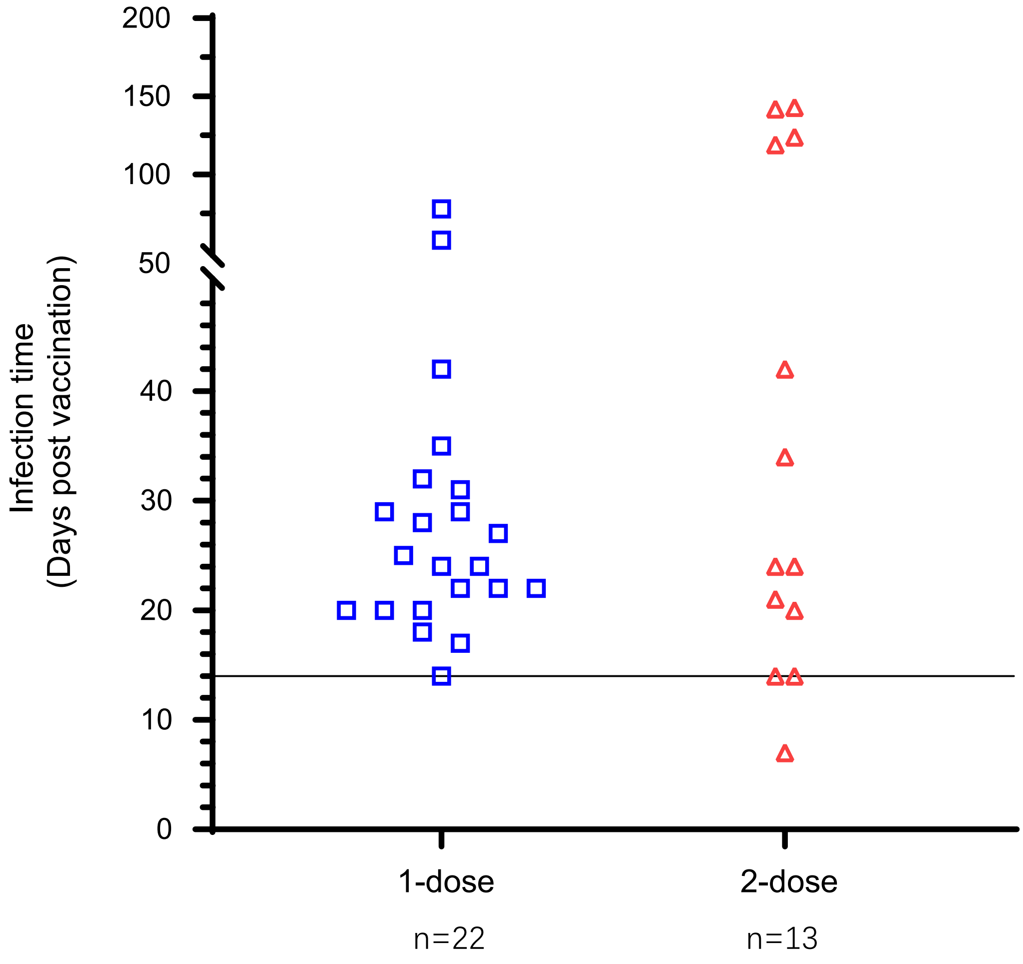


**Supplementary Figure 1. Time of breakthrough infection post-vaccination**

1-dose vaccine (blue square) and 2-dose vaccine (red triangle); the black solid line represents day 14. Source data are provided as a Source Data file.

Supp. Figure 2

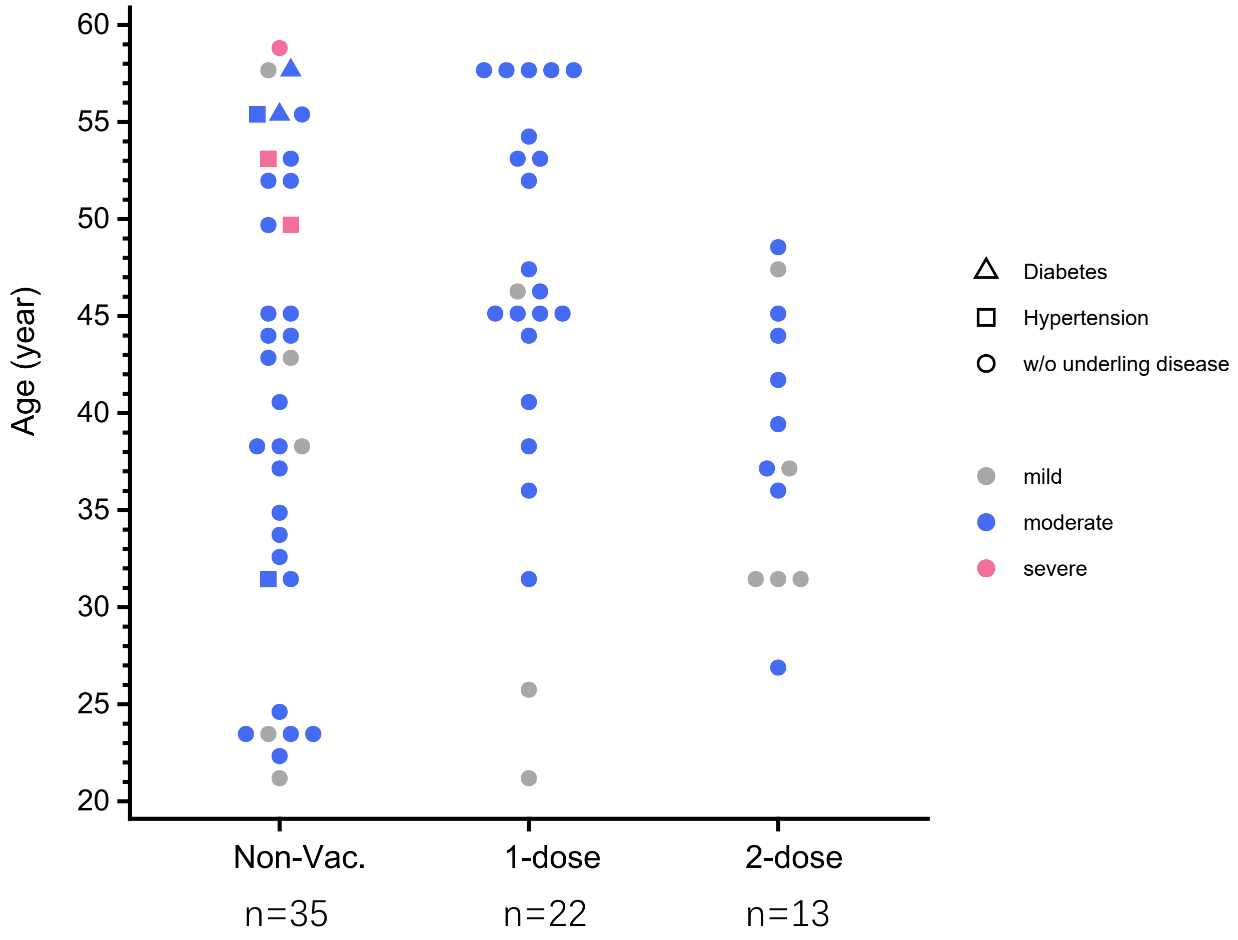


**Supplementary Figure 2. Age, clinical classification, and comorbidity distribution.** Clinical classification: mild (grey circle), moderate (blue circle), severe (red circle). Comorbidities: diabetes (triangle), hypertension (square), without comorbidities (circle). Source data are provided as a Source Data file.

Supp. Figure 3

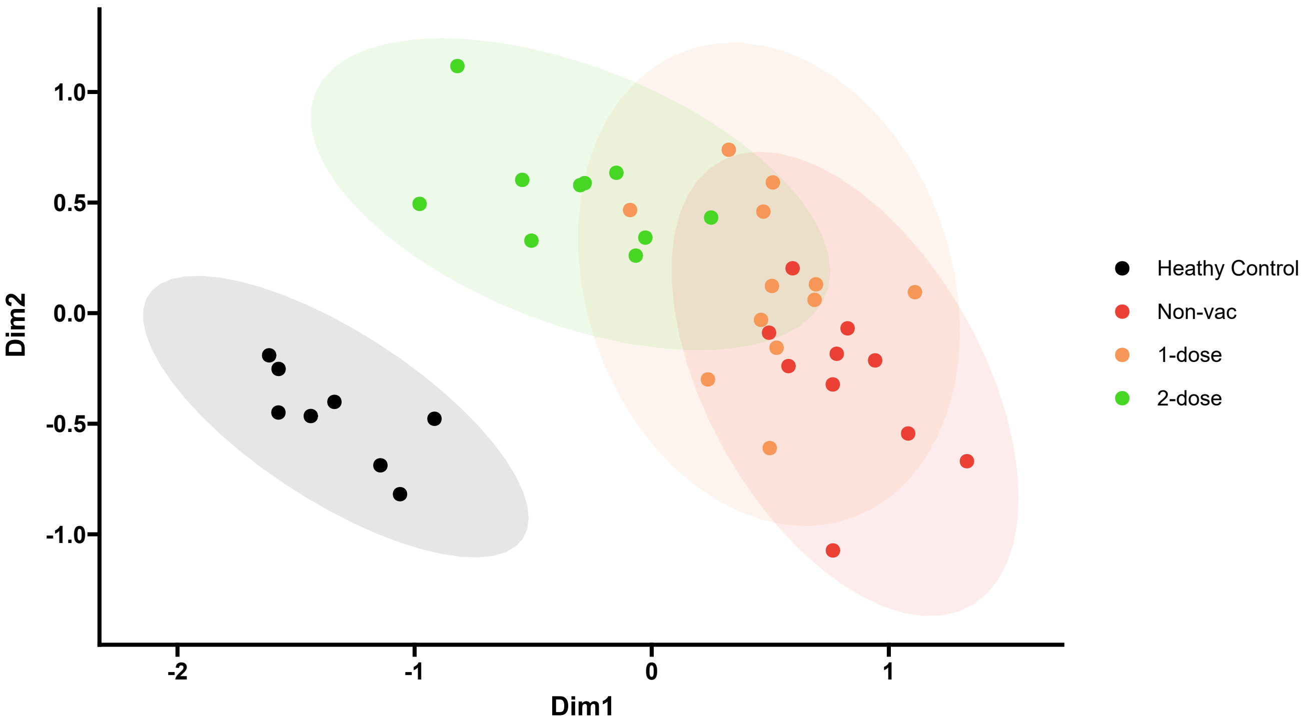


**Supplementary Figure 3. Principal component analysis of CPM values from transcriptome data.** Each dot corresponds to one RNA sequencing sample, and groups are indicated by different colours. Samples with similar gene expression profiles are more closely clustered.

Supp. Figure 4

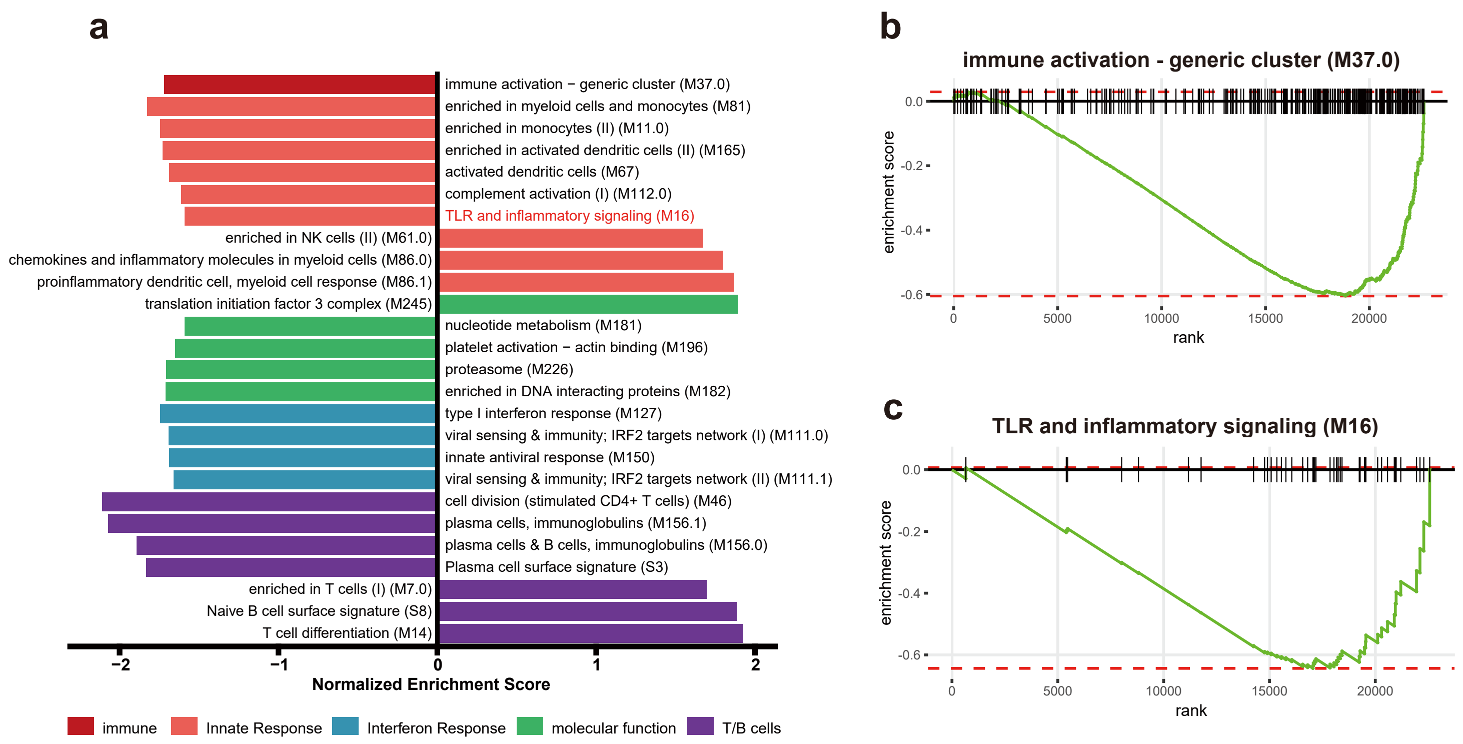


**Supplementary Figure 4. Gene set enrichment analysis. a** Immune-associated blood transcriptional modules (BTMs) were significantly enriched by DEGs in the nonvaccinated group versus the 2-dose group. Gene set enrichment analysis (GSEA) was performed within gene lists ranked by a metric score considering fold change and p value (p value was computed by using an exact test for the negative binomial distribution in edgeR package, which has strong parallels with Fisher's exact test, to assess differential expression). Detailed results of two important modules are shown: **b** immune activation - generic cluster (M37.0); **c** TLR and inflammatory signaling (M16).

**Supp. Figure 5**

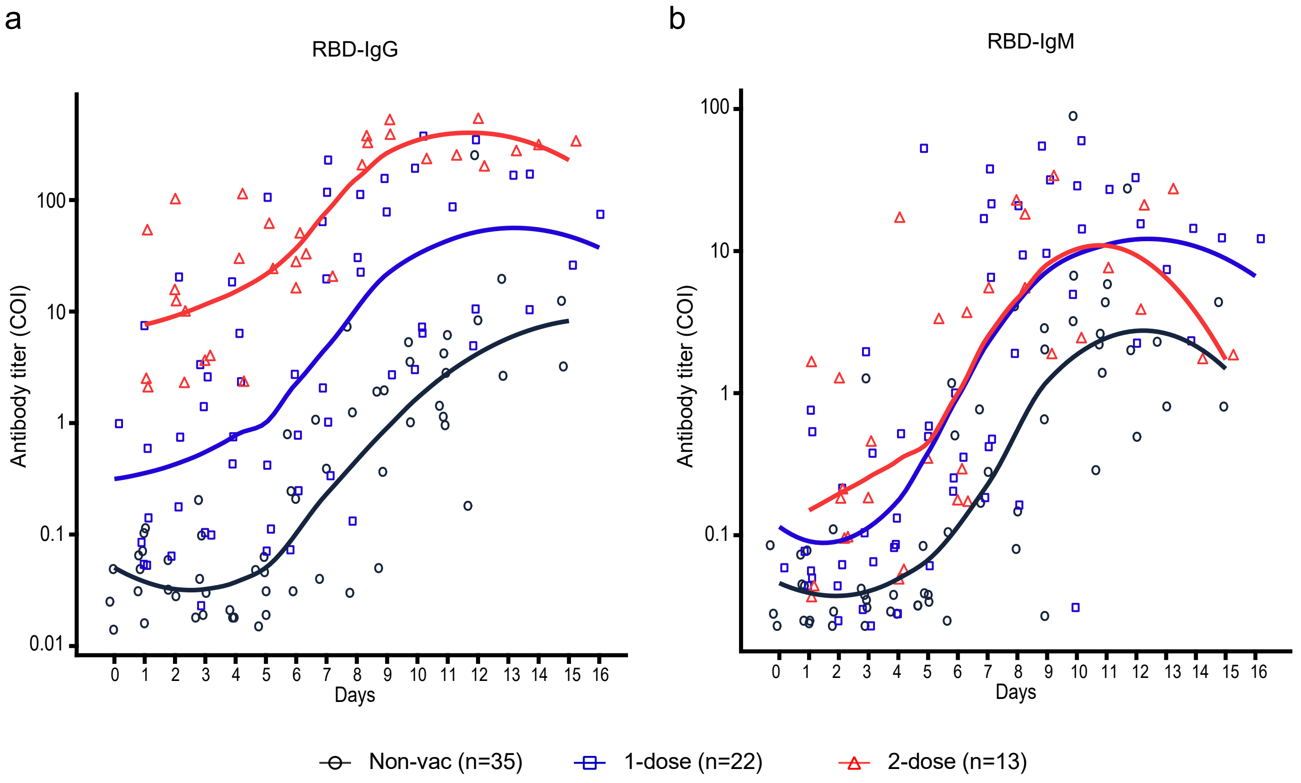


**Supplementary Figure 5. Trend of anti-RBD-specific IgM and IgG during the entire admission.** Nonvaccinated (black circle and black curve), 1-dose vaccine (blue square and square curve), 2-dose vaccine (red triangle and red curve). Source data are provided as a Source Data file.

Supp. Figure 6

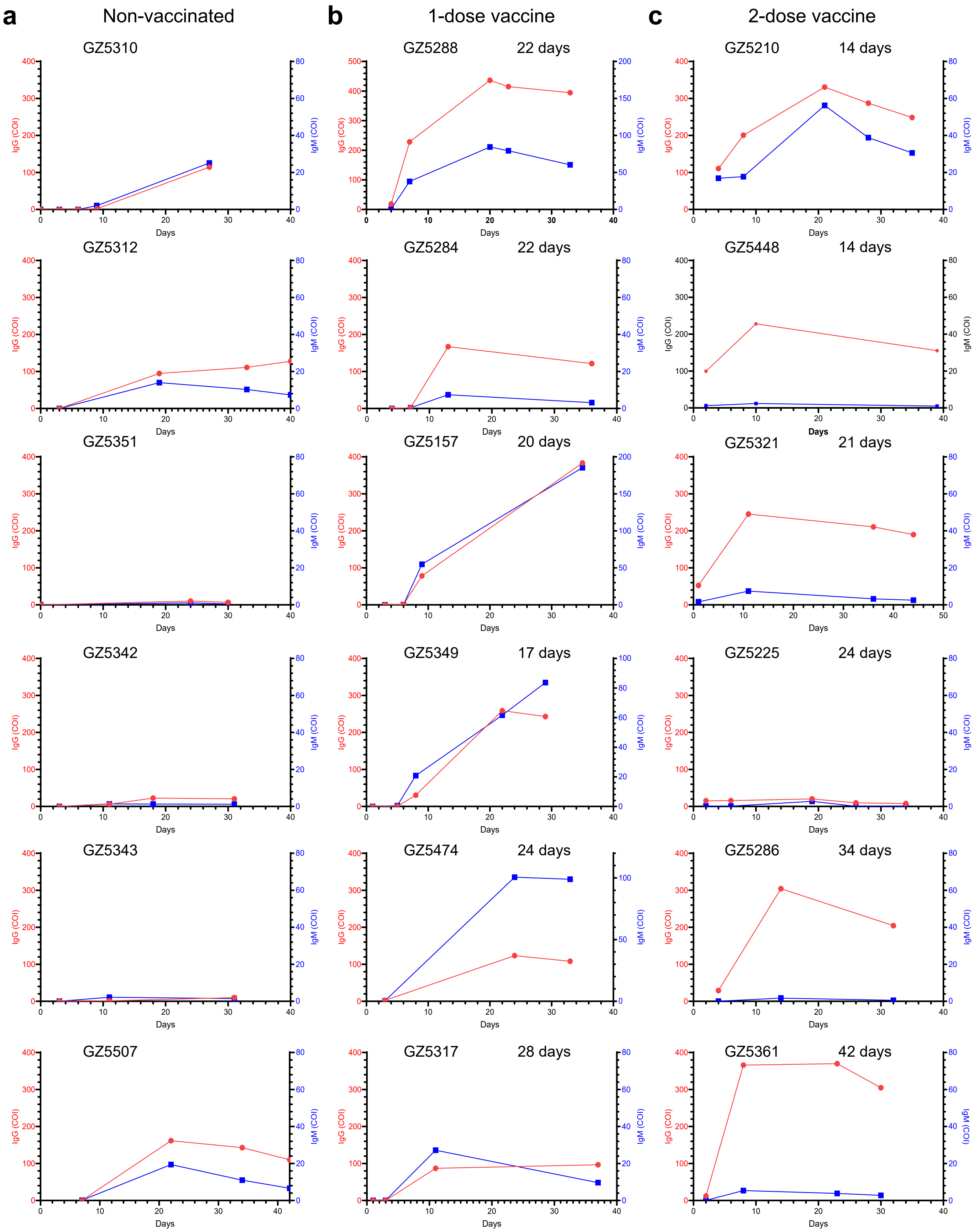


**Supplementary Figure 6. Features of Anti-RBD-specific IgM and IgG.**
**a** Nonvaccinated group. **b** 1-dose vaccine group. **c** 2-dose vaccine group.
See Figure 5. Source data are provided as a Source Data file.

# Supp Figure 7. Flow

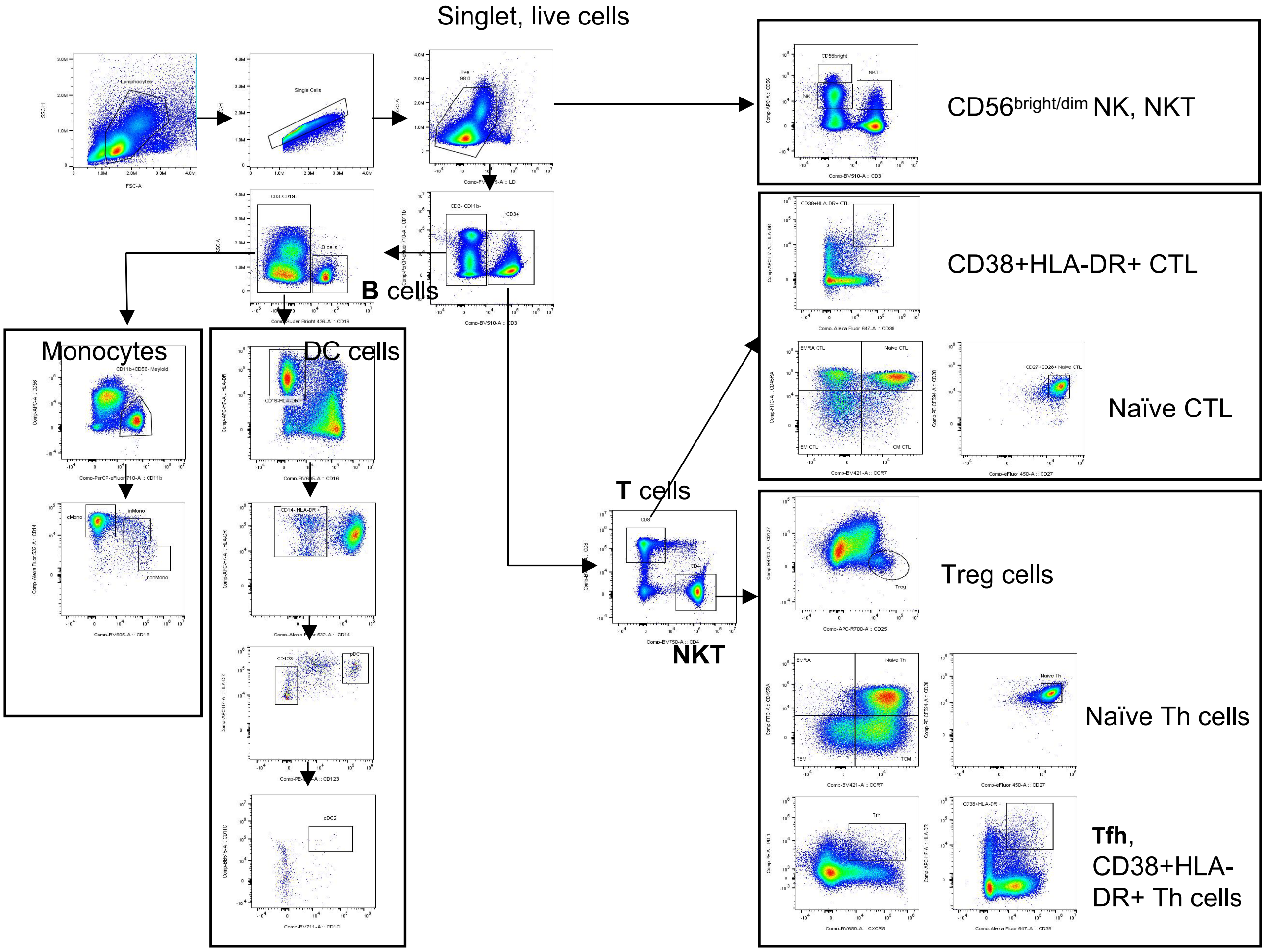


**Supplementary Figure 7. Gating logic of multi-parametric flow cytometry.**

Supp. Figure 8

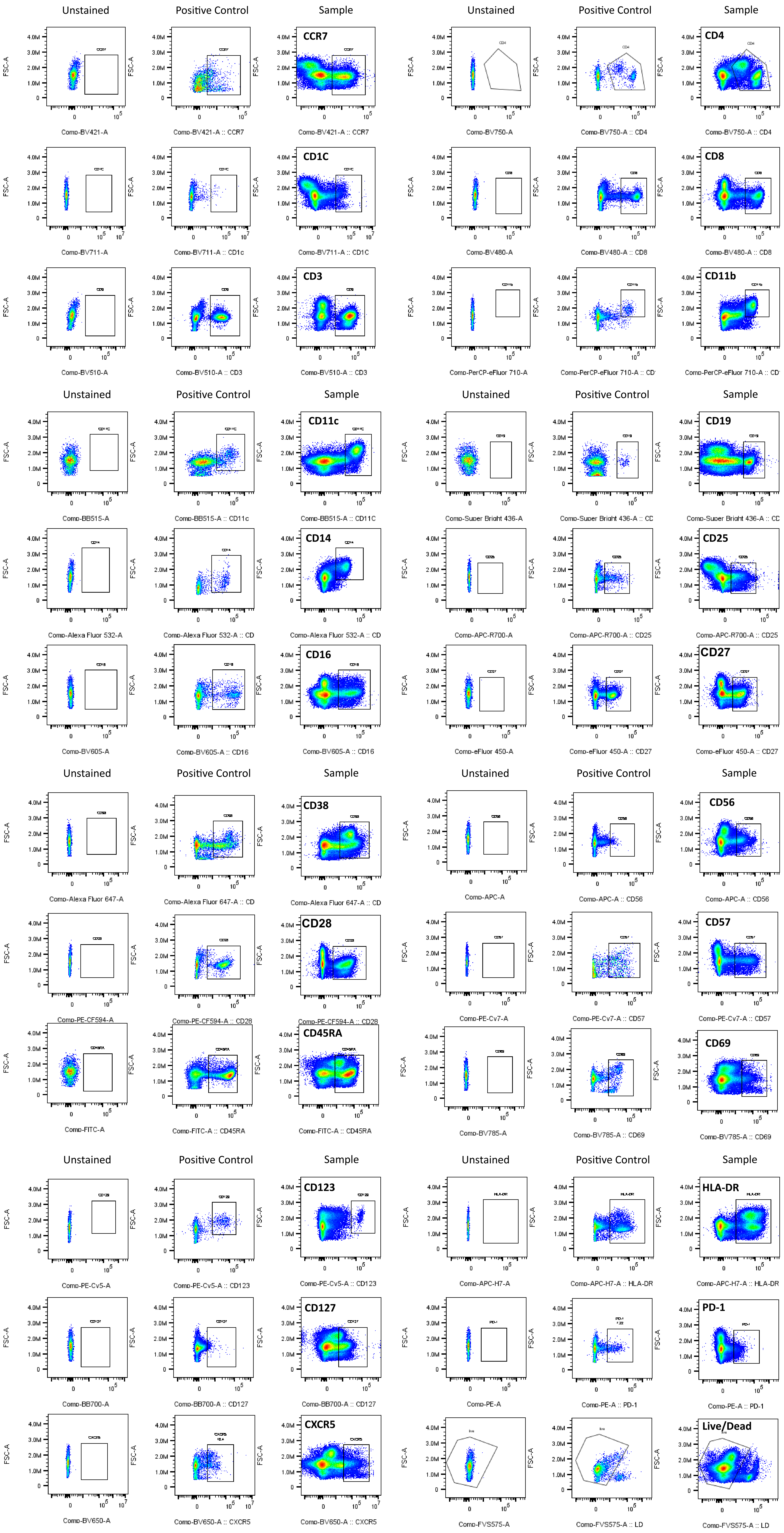


**Supplementary Figure 8. Staining control of multi-parametric flow cytometry.**

Supp. Figure 9

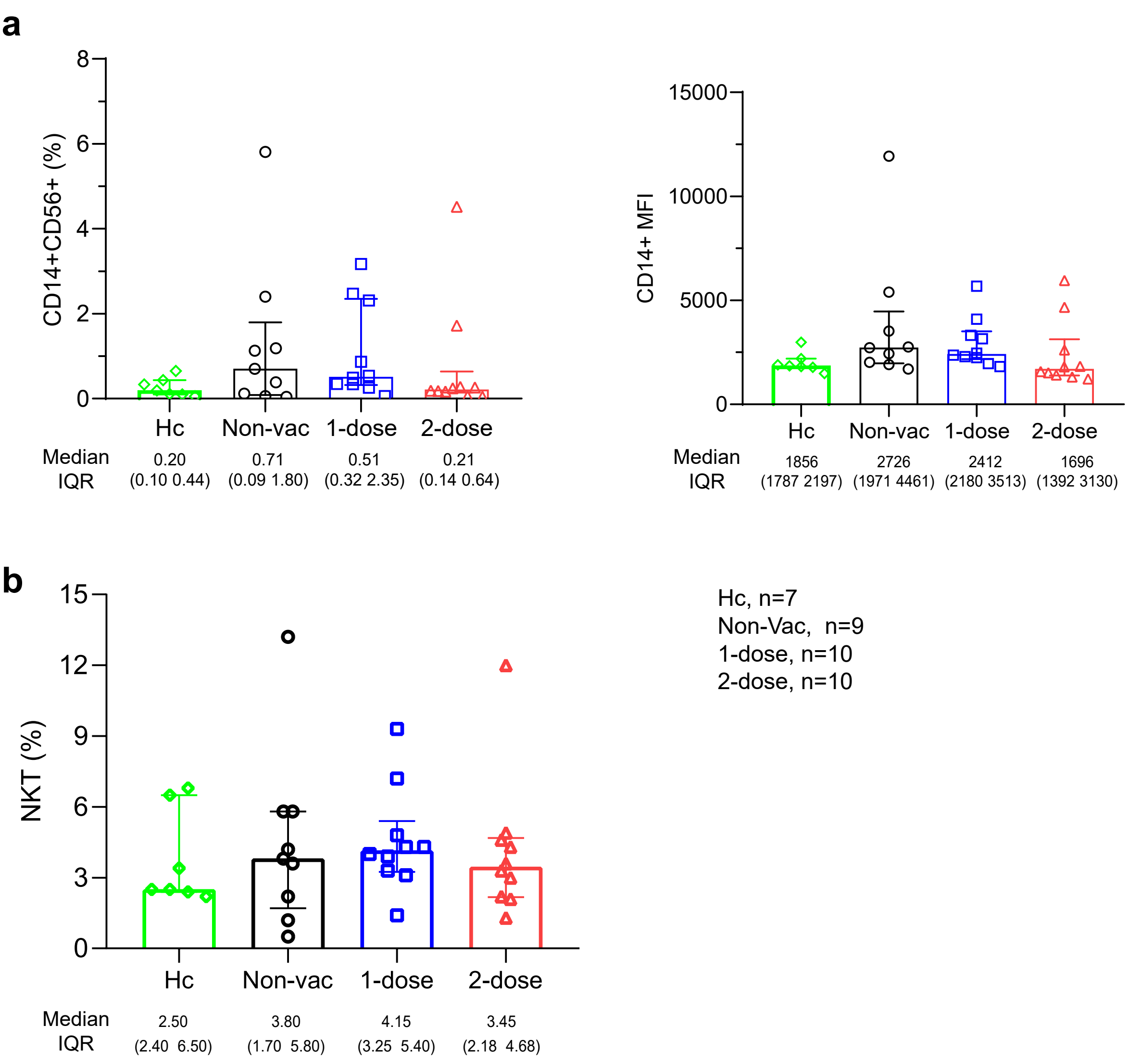


**Supplementary Figure 9. Flow cytometry analysis of CD14+CD56+NK, NKT, and CD14+ mean fluorescence intensity (MFI).** **a** CD14+CD56+NK cells and CD14+ MFI.
**b** NKT. Two-tailed p values (Kruskal-Wallis test) are indicated, and the data are the median (IQR). Comparisons without significant difference (p>0.05) are not shown.
Source data are provided as a Source Data file.

**Supplemental Table 1. Viral RNA level comparison from day 4 to day14 post symptom onset**

| | Non-vaccinated (n=35) | 2-dose vaccine (n=13) | P-value | 1-dose vaccine (n=22) | 2-dose vaccine (n=13) | P-value |
|---|---|---|---|---|---|---|
| Ct>30, n (%) | | | | | | |
| D4 | 0/35 (0) | 2/13 (15) | 0.069[a] | 1/22 (5) | 2/13 (15) | 0.541[a] |
| D6 | 2/35 (6) | 3/13 (23) | 0.115[a] | 2/22 (9) | 3/13 (23) | 0.337[a] |
| D8 | 4/35 (11) | 7/13 (54) | 0.004[a] | 3/22 (14) | 7/13 (54) | 0.020[a] |
| D10 | 15/35 (43) | 11/13 (85) | 0.010[b] | 9/22 (41) | 11/13 (85) | 0.016[a] |
| D12 | 23/35 (66) | 12/13 (92) | 0.081[a] | 12/22 (55) | 12/13 (92) | 0.027[a] |
| D14 | 24/35 (69) | 13/13 (100) | 0.023[a] | 16/22 (73) | 13/13 (100) | 0.064[a] |
| Ct>35, n (%) | | | | | | |
| D4 | 0/35 (0) | 1/13 (8) | 0.271[a] | 0/22 (0) | 1/13 (8) | 0.371[a] |
| D6 | 1/35 (3) | 1/13 (8) | 0.473[a] | 0/22 (0) | 1/13 (8) | 0.371[a] |
| D8 | 1/35 (3) | 2/13 (15) | 0.174[a] | 2/22 (9) | 2/13 (15) | 0.618[a] |
| D10 | 2/35 (6) | 7/13 (54) | 0.001[a] | 3/22 (14) | 7/13 (54) | 0.020[a] |
| D12 | 8/35 (23) | 10/13 (77) | 0.002[a] | 6/22 (27) | 10/13 (77) | 0.006[a] |
| D14 | 14/35 (40) | 13/13 (100) | <0.001[b] | 11/22 (50) | 13/13 (100) | 0.002[a] |

Two-tailed P values were calculated by [b]$\chi^2$ test or [a]Fisher's exact test, as appropriate.

Supplemental Table 2. Antibodies used for flow cytometry analysis.

| Marker | Fluorochrome | Manufacturer | Dilution μl/10^6 cell /100 μl | Clone | Cataloge No. |
|---|---|---|---|---|---|
| CD1c | BV711 | Biolegend | 2.0 | L161 | 331536 |
| CD8 | BV480 | BD Biosciences | 0.5 | RPA-T8 | 566121 |
| CD38 | Alexa Fluor 647 | Biolegend | 0.5 | HB-7 | 356632 |
| CD4 | BV750 | BD Biosciences | 0.5 | SK3 | 566355 |
| CD69 | BV786 | BD Biosciences | 1.0 | FN50 | 563834 |
| CD279(PD1) | PE | BD Biosciences | 10 | MIH4 | 557946 |
| CD57 | PE-Cy7 | Biolegend | 1.0 | HNK-1 | 359624 |
| HLA-DR | APC-H7 | BD Biosciences | 1.0 | G46-6 | 561358 |
| CD123 | PE-Cy5 | BD Biosciences | 5.0 | 9F5 | 551065 |
| CD27 | eFluor450 | eBioscience | 1.0 | O323 | 48-0279-42 |
| CD16 | BV605 | BD Biosciences | 1.0 | 3G8 | 563172 |
| CD45RA | FITC | BD Biosciences | 1.25 | HI100 | 555488 |
| CD127 | BB700 | BD Biosciences | 2.0 | HIL-7R-M21 | 566398 |
| CD14 | Alexa Fluor532 | eBioscience | 3.0 | 61D3 | 58-0149-41 |
| CD197(CCR7) | BV421 | BD Biosciences | 4.0 | 150503 | 562555 |
| CD19 | Super Bright436 | eBioscience | 4.0 | HIB19 | 62-0199-42 |
| CD56 | APC | BD Biosciences | 5.0 | B159 | 555518 |
| CD3 | BV510 | BD Biosciences | 3.0 | HIT3a | 564713 |
| CD11b | PerCP-eFluor 710 | eBioscience | 2.0 | ICRF44 | 46-0118-42 |
| FVS575V | BV570 | BD Biosciences | 0.2 | / | 565694 |
| CD25 | APC-R700 | BD Biosciences | 3.0 | 2A3 | 565106 |
| CD11c | BB515 | BD Biosciences | 1.0 | B-ly6 | 564490 |
| CD28 | PE-CF594 | BD Biosciences | 1.0 | CD28.2 | 562296 |
| CXCR5(CD185) | BV650 | BD Biosciences | 3.0 | RF8B2 | 740528 |
