# Supplementary material for: Clinical characteristics and immune profile alterations in vaccinated individuals with breakthrough Delta SARS-CoV-2 infections
Source: Nat Commun. 2022 Jul 9;13:3979. doi: 10.1038/s41467-022-31693-7 (PMC9271076; doi:10.1038/s41467-022-31693-7)
Supplement: Supplementary file 2 — Reporting Summary [file 41467_2022_31693_MOESM2_ESM.pdf]

nature portfolio

Corresponding author(s): Feng Li

Last updated by author(s): Jun 26, 2022

Reporting Summary

Nature Portfolio wishes to improve the reproducibility of the work that we publish. This form provides structure for consistency and transparency in reporting. For further information on Nature Portfolio policies, see our Editorial Policies and the Editorial Policy Checklist.

Statistics

For all statistical analyses, confirm that the following items are present in the figure legend, table legend, main text, or Methods section.

n/a	Confirmed	
☐	☒	The exact sample size (*n*) for each experimental group/condition, given as a discrete number and unit of measurement
☒	☐	A statement on whether measurements were taken from distinct samples or whether the same sample was measured repeatedly
☐	☒	The statistical test(s) used AND whether they are one- or two-sided<br>*Only common tests should be described solely by name; describe more complex techniques in the Methods section.*
☒	☐	A description of all covariates tested
☒	☐	A description of any assumptions or corrections, such as tests of normality and adjustment for multiple comparisons
☐	☒	A full description of the statistical parameters including central tendency (e.g. means) or other basic estimates (e.g. regression coefficient) AND variation (e.g. standard deviation) or associated estimates of uncertainty (e.g. confidence intervals)
☐	☒	For null hypothesis testing, the test statistic (e.g. *F*, *t*, *r*) with confidence intervals, effect sizes, degrees of freedom and *P* value noted<br>*Give P values as exact values whenever suitable.*
☒	☐	For Bayesian analysis, information on the choice of priors and Markov chain Monte Carlo settings
☒	☐	For hierarchical and complex designs, identification of the appropriate level for tests and full reporting of outcomes
☒	☐	Estimates of effect sizes (e.g. Cohen's *d*, Pearson's *r*), indicating how they were calculated

Our web collection on statistics for biologists contains articles on many of the points above.

Software and code

Policy information about availability of computer code

Data collection	Clinical characteristic, laboratory findings were collected from the hospital information system (HIS) and laboratory information system (LIS) of the hospital. The flow cytometric data were collected on a spectral flow cytometry (Cytek NL-CLC, Cytek Biosciences, USA). RNA-sequencing data were collected on MGISEQ-2000 platform (MGI, Shenzhen, P. R. China) in a 100 double-end sequencing method. inflammatory cytokines data were collected on Inflammation multiplex platform (Cat. v.3022, Olink Proteomics AB, Uppsala, Sweden).
Data analysis	Continuous variables were expressed as median (interquartile range, IQR). Categorical variables were summarized as the counts and percentages in each category. Mann-Whitney U tests, ANOVA tests or Kruskal-Wallis tests were applied to continuous variables as appropriate, chi-square test or Fisher's exact test were applied to categorical variables as appropriate, log-rank (Mantel–Cox) test was applied to virus RNA clearance, p<0.05 was considered statistically significant. Statistical analysis was performed with IBM SPSS Statistics 25. Graphic representations were performed with GraphPad Prism 8.0.1 software. Full-spectrum heatmap of 92 inflammatory cytokines were performed with pheatmap (v.1.0.12) packages. RNA-sequencing data processing and analysis were performed with fastp, STAR, featureCounts, edgeR (v.3.32.1) packages, fgsea (v.1.16.0) packages. The flow cytometric data were analyzed using FlowJo software V10.7.1.

For manuscripts utilizing custom algorithms or software that are central to the research but not yet described in published literature, software must be made available to editors and reviewers. We strongly encourage code deposition in a community repository (e.g. GitHub). See the Nature Portfolio guidelines for submitting code & software for further information.

Data

Policy information about availability of data

All manuscripts must include a data availability statement. This statement should provide the following information, where applicable:

- Accession codes, unique identifiers, or web links for publicly available datasets
- A description of any restrictions on data availability
- For clinical datasets or third party data, please ensure that the statement adheres to our policy

Data availability
Source data for all tables and figures are provided with this paper. The raw RNA-sequencing data reported in this paper have been deposited in the Genome Sequence Archive for Human under accession code HRA002352 and are accessible at https://ngdc.cncb.ac.cn/gsa-human/s/S0u3Oey2 .

Field-specific reporting

Please select the one below that is the best fit for your research. If you are not sure, read the appropriate sections before making your selection.

☒ Life sciences ☐ Behavioural & social sciences ☐ Ecological, evolutionary & environmental sciences

For a reference copy of the document with all sections, see nature.com/documents/nr-reporting-summary-flat.pdf

Life sciences study design

All studies must disclose on these points even when the disclosure is negative.

Sample size	In this retrospective study, a total of 157 patients confirmed by the local CDC from the outbreak of SARS-CoV-2 Delta VOC in Guangzhou were included in the study. According to selection criteria: excluded age < 18 26 cases, age ≥ 60 52 cases, non-vaccinated 35 cases were included; 1-dose (Immunization time≥ 14 days) 22 cases were included; 2-dose 13 cases were included. Finally, a total of 70 patients were included in the study cohort. This study was a retrospective, some data had fewer statistical points, such as 0 w: week 0, days 0-5. Fortunately, as a designated treatment hospital for COVID-19, we carried out a complete tracking of the patient's clinical characteristics, antibody levels and SARS-CoV-2 viral RNA. In addition, we had complete scientific research sampling system, meeting the needs of samples. All the statistics involved in this study indicate the number of samples, meeting the relevant statistical requirements.
Data exclusions	As only individuals between 18 to 60 years old were recommended to be vaccinated before the Delta VOC outbreak in Guangzhou, 26 patients younger than 18-year and 52 patients aged 60 or older were excluded for subsequent analysis.
Replication	This study is a real-world observation. All the data about clinical measurement, such as IgG levels, SARS-CoV-2 viral RNA, was collected from the Laboratory Information System (LIS), which was measured by instruments and reagents approved by China FDA. Clinical characteristics were collected from Hospital Information System (HIS). RNA-sequencing, inflammatory cytokines measurement and flow cytometry were performed independently, these results were repeatable.
Randomization	This is a retrospective study. Most clinical data is based on a real-world observation, randomization is not applicable in this regard. For RNA-sequencing, inflammatory cytokines measurement and flow cytometry, we all performed randomization assays.
Blinding	This is a retrospective study, the experimental design is based on real-world observations. Therefore, blinding is not applicable to the study.

Behavioural & social sciences study design

All studies must disclose on these points even when the disclosure is negative.

Study description	*Briefly describe the study type including whether data are quantitative, qualitative, or mixed-methods (e.g. qualitative cross-sectional, quantitative experimental, mixed-methods case study).*
Research sample	*State the research sample (e.g. Harvard university undergraduates, villagers in rural India) and provide relevant demographic information (e.g. age, sex) and indicate whether the sample is representative. Provide a rationale for the study sample chosen. For studies involving existing datasets, please describe the dataset and source.*
Sampling strategy	*Describe the sampling procedure (e.g. random, snowball, stratified, convenience). Describe the statistical methods that were used to predetermine sample size OR if no sample-size calculation was performed, describe how sample sizes were chosen and provide a rationale for why these sample sizes are sufficient. For qualitative data, please indicate whether data saturation was considered, and what criteria were used to decide that no further sampling was needed.*
Data collection	*Provide details about the data collection procedure, including the instruments or devices used to record the data (e.g. pen and paper, computer, eye tracker, video or audio equipment) whether anyone was present besides the participant(s) and the researcher, and whether the researcher was blind to experimental condition and/or the study hypothesis during data collection.*
Timing	*Indicate the start and stop dates of data collection. If there is a gap between collection periods, state the dates for each sample*

Timing	*cohort.*
Data exclusions	*If no data were excluded from the analyses, state so OR if data were excluded, provide the exact number of exclusions and the rationale behind them, indicating whether exclusion criteria were pre-established.*
Non-participation	*State how many participants dropped out/declined participation and the reason(s) given OR provide response rate OR state that no participants dropped out/declined participation.*
Randomization	*If participants were not allocated into experimental groups, state so OR describe how participants were allocated to groups, and if allocation was not random, describe how covariates were controlled.*

Ecological, evolutionary & environmental sciences study design

All studies must disclose on these points even when the disclosure is negative.

Study description	*Briefly describe the study. For quantitative data include treatment factors and interactions, design structure (e.g. factorial, nested, hierarchical), nature and number of experimental units and replicates.*
Research sample	*Describe the research sample (e.g. a group of tagged Passer domesticus, all Stenocereus thurberi within Organ Pipe Cactus National Monument), and provide a rationale for the sample choice. When relevant, describe the organism taxa, source, sex, age range and any manipulations. State what population the sample is meant to represent when applicable. For studies involving existing datasets, describe the data and its source.*
Sampling strategy	*Note the sampling procedure. Describe the statistical methods that were used to predetermine sample size OR if no sample-size calculation was performed, describe how sample sizes were chosen and provide a rationale for why these sample sizes are sufficient.*
Data collection	*Describe the data collection procedure, including who recorded the data and how.*
Timing and spatial scale	*Indicate the start and stop dates of data collection, noting the frequency and periodicity of sampling and providing a rationale for these choices. If there is a gap between collection periods, state the dates for each sample cohort. Specify the spatial scale from which the data are taken*
Data exclusions	*If no data were excluded from the analyses, state so OR if data were excluded, describe the exclusions and the rationale behind them, indicating whether exclusion criteria were pre-established.*
Reproducibility	*Describe the measures taken to verify the reproducibility of experimental findings. For each experiment, note whether any attempts to repeat the experiment failed OR state that all attempts to repeat the experiment were successful.*
Randomization	*Describe how samples/organisms/participants were allocated into groups. If allocation was not random, describe how covariates were controlled. If this is not relevant to your study, explain why.*
Blinding	*Describe the extent of blinding used during data acquisition and analysis. If blinding was not possible, describe why OR explain why blinding was not relevant to your study.*

Did the study involve field work? ☐ Yes ☐ No

Field work, collection and transport

Field conditions	*Describe the study conditions for field work, providing relevant parameters (e.g. temperature, rainfall).*
Location	*State the location of the sampling or experiment, providing relevant parameters (e.g. latitude and longitude, elevation, water depth).*
Access & import/export	*Describe the efforts you have made to access habitats and to collect and import/export your samples in a responsible manner and in compliance with local, national and international laws, noting any permits that were obtained (give the name of the issuing authority, the date of issue, and any identifying information).*
Disturbance	*Describe any disturbance caused by the study and how it was minimized.*

Reporting for specific materials, systems and methods

We require information from authors about some types of materials, experimental systems and methods used in many studies. Here, indicate whether each material, system or method listed is relevant to your study. If you are not sure if a list item applies to your research, read the appropriate section before selecting a response.

Materials & experimental systems

n/a	Involved in the study
☐	☒ Antibodies
☒	☐ Eukaryotic cell lines
☒	☐ Palaeontology and archaeology
☒	☐ Animals and other organisms
☐	☒ Human research participants
☐	☒ Clinical data
☒	☐ Dual use research of concern

Methods

n/a	Involved in the study
☒	☐ ChIP-seq
☐	☒ Flow cytometry
☒	☐ MRI-based neuroimaging

Antibodies

Antibodies used

Marker	Fluorochrome	Manufacturer	Catalogue No.	Dilution(µl/10^6 cell /100 µl)	Clone
CD1c	BV711	Biolegend	331536	2.0	L161
CD8	BV480	BD Biosciences	566121	0.5	RPA-T8
CD38	Alexa Fluor 647	Biolegend	356632	0.5	HB-7
CD4	BV750	BD Biosciences	566355	0.5	SK3
CD69	BV786	BD Biosciences	563834	1.0	FN50
CD279(PD1	PE	BD Biosciences	557946	10	MIH4
CD57	PE-Cy7	Biolegend	359624	1.0	HNK-1
HLA-DR	APC-H7	BD Biosciences	561358	1.0	G46-6
CD123	PE-Cy5	BD Biosciences	551065	5.0	9F5
CD27	eFluor450	eBioscience	48-0279-42	1.0	O323
CD16	BV605	BD Biosciences	563172	1.0	3G8
CD45RA	FITC	BD Biosciences	555488	1.25	HI100
CD127	BB700	BD Biosciences	566398	2.0	HIL-7R-M21
CD14	Alexa Fluor532	eBioscience	58-0149-41	3.0	61D3
CD197(CCR7	BV421	BD Biosciences	562555	4.0	150503
CD19	Super Bright436	eBioscience	62-0199-42	4.0	HIB19
CD56	APC	BD Biosciences	555518	5.0	B159
CD3	BV510	BD Biosciences	564713	3.0	HIT3a
CD11b	PerCP-eFluor710	eBioscience	46-0118-42	2.0	ICRF44
FVS575V	BV570	BD Biosciences	565694	0.2	/
CD25	APC-R700	BD Biosciences	565106	3.0	2A3
CD11c	BB515	BD Biosciences	564490	1.0	B-ly6
CD28	PE-CF594	BD Biosciences	562296	1.0	CD28.2
CXCR5(CD185)	BV650	BD Biosciences	740528	3.0	RF8B2

Validation

CD1c: host species mouse; application: Flow Cytometry, Western Blotting, Immunofluorescence, Immunocytochemistry; reactivity: human
CD8: host species mouse; application: Flow Cytometry; reactivity: human
CD38: host species mouse; application: Flow Cytometry, Western Blotting, Immunofluorescence, Immunocytochemistry; reactivity: human
CD4: host species mouse; application: Flow Cytometry; reactivity: human
CD69: host species mouse; application: Flow Cytometry; reactivity: human
CD279(PD1): host species MIH4; application: Flow Cytometry; reactivity: human
CD57: host species mouse; application: Flow Cytometry, Western Blotting, Immunofluorescence, Immunocytochemistry; reactivity: human
HLA-DR: host species mouse; application: Flow Cytometry; reactivity: human
CD123: host species mouse; application: Flow Cytometry; reactivity: human
CD27: host species mouse; application: Flow Cytometry; reactivity: human
CD16: host species mouse; application: Flow Cytometry; reactivity: human, baboon, cynomolgus, rhesus.
CD45R: host species mouse; application: Flow Cytometry; reactivity: human
CD127: host species mouse; application: Flow Cytometry; reactivity: human
CD14: host species mouse; application: Flow Cytometry, Immunofluorescence, Immunocytochemistry; reactivity: human
CD197(CCR7): host species mouse; application: Flow Cytometry; reactivity: human
CD19: host species mouse; application: Flow Cytometry, Immunofluorescence, Immunocytochemistry; reactivity: human
CD56: host species mouse; application: Flow Cytometry; reactivity: human
CD3: host species mouse; application: Flow Cytometry; reactivity: human
CD11b: host species mouse; application: Flow Cytometry; reactivity: human
CD25: host species mouse; application: Flow Cytometry; reactivity: human
CD11c: host species mouse; application: Flow Cytometry; reactivity: human
CD28: host species mouse; application: Flow Cytometry; reactivity: human
CXCR5(CD185): host species rat; application: Flow Cytometry; reactivity: human.

Above validation is available from the commercial providers.

March 2021

Eukaryotic cell lines

Policy information about cell lines

Cell line source(s)	NA
Authentication	NA
Mycoplasma contamination	NA
Commonly misidentified lines (See ICLAC register)	NA

Palaeontology and Archaeology

Specimen provenance	NA
Specimen deposition	NA
Dating methods	NA

☐ Tick this box to confirm that the raw and calibrated dates are available in the paper or in Supplementary Information.

Ethics oversight	NA

Note that full information on the approval of the study protocol must also be provided in the manuscript.

Animals and other organisms

Policy information about studies involving animals; ARRIVE guidelines recommended for reporting animal research

Laboratory animals	NA
Wild animals	NA
Field-collected samples	NA
Ethics oversight	NA

Note that full information on the approval of the study protocol must also be provided in the manuscript.

Human research participants

Policy information about studies involving human research participants

Population characteristics	All patients were from Guangzhou Eighth People's Hospital, Guangzhou Medical University from May 21, 2021 (the first case was officially confirmed to be infected with SARS-CoV-2 Delta VOC) to July 9, 2021 (the discharge day of all local patients). All patients were confirmed to be linked to a single Delta VOC origin by epidemiological evidence and viral genome sequencing. A total of 157 individuals were included in this study. According to selection criteria: excluded age < 18 26 cases, age ≥ 60 52 cases, non-vaccinated 35 cases were included. Twelve of 35 (34%) patients were male in the non-vaccinated group. The median age of the non-vaccinated group was 43 (21 to 59) years old; 1-dose (Immunization time≥ 14 days) 22 cases were included. 6 of 22 (27%) were male in the 1-dose vaccine group. The median age of 1-dose group was 46 (21 to 58) years old; 2-dose 13 cases were included, 7 of 13 (53%) were male in the 2-dose vaccine group. The median age of the 2-dose group was 39 (27 to 58) years old. Among these patients, supportive treatment was used for mild and moderate patients, assisted ventilation was used for severe patients, and short-dose methylprednisolone suppressed excessive immune inflammation responses.
Recruitment	The Guangzhou Eighth People's Hospital was a designated treatment hospital for COVID-19 in Guangzhou. All patients from May 21, 2021 (the first case was officially confirmed to be infected with SARS-CoV-2 Delta VOC) to July 9, 2021 (the discharge day of all local patients) were at the Guangzhou Eighth People's Hospital. All samples were collected with the consent of the patients and signed informed consent.
Ethics oversight	The study was approved by Guangzhou Eighth People's Hospital Ethics Committee (No. 202001134 and 202115202). Written informed consents were obtained from all patients.

Note that full information on the approval of the study protocol must also be provided in the manuscript.

Clinical data

Policy information about clinical studies

All manuscripts should comply with the ICMJE guidelines for publication of clinical research and a completed CONSORT checklist must be included with all submissions.

Clinical trial registration: This study is a real-world observation, is not registered online.

Study protocol: This study is a retrospective observation.

Data collection: Clinical characteristic, laboratory findings were collected from the hospital information system and laboratory information system of the hospital. All diagnoses were made based on the Guidelines for the Diagnosis and Treatment of Novel Coronavirus Infection produced by the Chinese National Health Commission (Trial Version 8). Clinical characteristic included clinical classification (mild, moderate, severe), comorbidities (hypertension, diabetes, chronic heart disease, liver disease, lung disease and thyroid) and symptoms (fever, cough, sputum, sore throat, dyspnea, vomit, headache, diarrhea, fatigue.). Demographics included sex, age, smoking history.

Outcomes: NA

Dual use research of concern

Policy information about dual use research of concern

Hazards

Could the accidental, deliberate or reckless misuse of agents or technologies generated in the work, or the application of information presented in the manuscript, pose a threat to:

No	Yes	
☐	☐	Public health
☐	☐	National security
☐	☐	Crops and/or livestock
☐	☐	Ecosystems
☐	☐	Any other significant area

Experiments of concern

Does the work involve any of these experiments of concern:

No	Yes	
☐	☐	Demonstrate how to render a vaccine ineffective
☐	☐	Confer resistance to therapeutically useful antibiotics or antiviral agents
☐	☐	Enhance the virulence of a pathogen or render a nonpathogen virulent
☐	☐	Increase transmissibility of a pathogen
☐	☐	Alter the host range of a pathogen
☐	☐	Enable evasion of diagnostic/detection modalities
☐	☐	Enable the weaponization of a biological agent or toxin
☐	☐	Any other potentially harmful combination of experiments and agents

ChIP-seq

Data deposition

☐ Confirm that both raw and final processed data have been deposited in a public database such as GEO.

☐ Confirm that you have deposited or provided access to graph files (e.g. BED files) for the called peaks.

Data access links
May remain private before publication.: *For "Initial submission" or "Revised version" documents, provide reviewer access links. For your "Final submission" document, provide a link to the deposited data.*

Files in database submission: *Provide a list of all files available in the database submission.*

Genome browser session
(e.g. UCSC): *Provide a link to an anonymized genome browser session for "Initial submission" and "Revised version" documents only, to enable peer review. Write "no longer applicable" for "Final submission" documents.*

Methodology

Replicates	*Describe the experimental replicates, specifying number, type and replicate agreement.*
Sequencing depth	*Describe the sequencing depth for each experiment, providing the total number of reads, uniquely mapped reads, length of reads and whether they were paired- or single-end.*
Antibodies	*Describe the antibodies used for the ChIP-seq experiments; as applicable, provide supplier name, catalog number, clone name, and lot number.*
Peak calling parameters	*Specify the command line program and parameters used for read mapping and peak calling, including the ChIP, control and index files used.*
Data quality	*Describe the methods used to ensure data quality in full detail, including how many peaks are at FDR 5% and above 5-fold enrichment.*
Software	*Describe the software used to collect and analyze the ChIP-seq data. For custom code that has been deposited into a community repository, provide accession details.*

Flow Cytometry

Plots

Confirm that:

☒ The axis labels state the marker and fluorochrome used (e.g. CD4-FITC).

☒ The axis scales are clearly visible. Include numbers along axes only for bottom left plot of group (a 'group' is an analysis of identical markers).

☒ All plots are contour plots with outliers or pseudocolor plots.

☒ A numerical value for number of cells or percentage (with statistics) is provided.

Methodology

Sample preparation	Multi-parametric flow cytometry was used for phenotypic analysis of PBMCs from patients and healthy donors. Briefly, 1×106 ~2×106 PBMCs were washed twice with DPBS (BI, 02-023-1A) and stained with FVS575V for 20min at RT in the dark for live/dead. Then cells were washed twice (400×g, 5 min, 4?) with FACS buffer (2% FBS/PBS) and stained with CXCR5-BV650 and CCR7-BV421 for 10min at RT in the dark. Next, other 21 kinds of surface receptor staining mix were incubated with cells for another 30min at RT in the dark. After incubation, cells were washed with FACS buffer (400×g, 5 min, 4?) and resuspended in 200ul FACS buffer.
Instrument	Cytek NL-CLC, Cytek Biosciences, USA
Software	FlowJo software, V10.7.1 (Tree Star, USA)
Cell population abundance	No sorting was used in this study.
Gating strategy	We established a 24-color reference library by detecting unstained samples tube and single-positive staining tubes to distinguish positive and negative cell populations. In the FSC/SSC state, polygonal gate circles the immune cell populations, remove adherent cells to obtain a single cell population, and remove dead cells by FVS575V staining, polygonal gate circles live cells. CD3+ T cells and CD3-CD11b- cells, NK, NKT were obtained in the live cell gate. CD4+ T cells, CD8+ T cells, Treg cells, Th cells and the activated state cell populations were analyzed in the CD3+ T cell population. B cells, DCs and monocytes were analyzed in the CD3-CD11b-cell population.

☒ Tick this box to confirm that a figure exemplifying the gating strategy is provided in the Supplementary Information.

Magnetic resonance imaging

Experimental design

Design type	*Indicate task or resting state; event-related or block design.*
Design specifications	*Specify the number of blocks, trials or experimental units per session and/or subject, and specify the length of each trial or block (if trials are blocked) and interval between trials.*
Behavioral performance measures	*State number and/or type of variables recorded (e.g. correct button press, response time) and what statistics were used to establish that the subjects were performing the task as expected (e.g. mean, range, and/or standard deviation across subjects).*

Acquisition

Imaging type(s) — *Specify: functional, structural, diffusion, perfusion.*

Field strength — *Specify in Tesla*

Sequence & imaging parameters — *Specify the pulse sequence type (gradient echo, spin echo, etc.), imaging type (EPI, spiral, etc.), field of view, matrix size, slice thickness, orientation and TE/TR/flip angle.*

Area of acquisition — *State whether a whole brain scan was used OR define the area of acquisition, describing how the region was determined.*

Diffusion MRI ☐ Used ☐ Not used

Preprocessing

Preprocessing software — *Provide detail on software version and revision number and on specific parameters (model/functions, brain extraction, segmentation, smoothing kernel size, etc.).*

Normalization — *If data were normalized/standardized, describe the approach(es): specify linear or non-linear and define image types used for transformation OR indicate that data were not normalized and explain rationale for lack of normalization.*

Normalization template — *Describe the template used for normalization/transformation, specifying subject space or group standardized space (e.g. original Talairach, MNI305, ICBM152) OR indicate that the data were not normalized.*

Noise and artifact removal — *Describe your procedure(s) for artifact and structured noise removal, specifying motion parameters, tissue signals and physiological signals (heart rate, respiration).*

Volume censoring — *Define your software and/or method and criteria for volume censoring, and state the extent of such censoring.*

Statistical modeling & inference

Model type and settings — *Specify type (mass univariate, multivariate, RSA, predictive, etc.) and describe essential details of the model at the first and second levels (e.g. fixed, random or mixed effects; drift or auto-correlation).*

Effect(s) tested — *Define precise effect in terms of the task or stimulus conditions instead of psychological concepts and indicate whether ANOVA or factorial designs were used.*

Specify type of analysis: ☐ Whole brain ☐ ROI-based ☐ Both

Statistic type for inference (See Eklund et al. 2016) — *Specify voxel-wise or cluster-wise and report all relevant parameters for cluster-wise methods.*

Correction — *Describe the type of correction and how it is obtained for multiple comparisons (e.g. FWE, FDR, permutation or Monte Carlo).*

Models & analysis

n/a	Involved in the study
☐	☐ Functional and/or effective connectivity
☐	☐ Graph analysis
☐	☐ Multivariate modeling or predictive analysis

Functional and/or effective connectivity — *Report the measures of dependence used and the model details (e.g. Pearson correlation, partial correlation, mutual information).*

Graph analysis — *Report the dependent variable and connectivity measure, specifying weighted graph or binarized graph, subject- or group-level, and the global and/or node summaries used (e.g. clustering coefficient, efficiency, etc.).*

Multivariate modeling and predictive analysis — *Specify independent variables, features extraction and dimension reduction, model, training and evaluation metrics.*
